# Essential role of the TFIID subunit TAF4 in murine embryogenesis and embryonic stem cell differentiation

**DOI:** 10.1038/ncomms11063

**Published:** 2016-03-30

**Authors:** Diana Langer, Igor Martianov, Daniel Alpern, Muriel Rhinn, Céline Keime, Pascal Dollé, Gabrielle Mengus, Irwin Davidson

**Affiliations:** 1 Department of Functional Genomics and Cancer, Institut de Génétique et de Biologie Moléculaire et Cellulaire, CNRS/INSERM/ULP, 1 Rue Laurent Fries, 67404 Illkirch, France; 2 L'École polytechnique fédérale de Lausanne, Route Cantonale, 1015 Lausanne, Switzerland; 3 Department of Development and Stem Cells, Institut de Génétique et de Biologie Moléculaire et Cellulaire, CNRS/INSERM/ULP, 1 Rue Laurent Fries, 67404 Illkirch, France

## Abstract

TAF4 (TATA-binding protein-associated factor 4) and its paralogue TAF4b are components of the TFIID core module. We inactivated the murine *Taf4a* gene to address Taf4 function during embryogenesis. Here we show that *Taf4a*^−/−^ embryos survive until E9.5 where primary germ layers and many embryonic structures are identified showing Taf4 is dispensable for their specification. In contrast, Taf4 is required for correct patterning of the trunk and anterior structures, ventral morphogenesis and proper heart positioning. Overlapping expression of *Taf4a* and *Taf4b* during embryogenesis suggests their redundancy at early stages. In agreement with this, *Taf4a*^−/−^ embryonic stem cells (ESCs) are viable and comprise Taf4b-containing TFIID. Nevertheless, *Taf4a*^−/−^ ESCs do not complete differentiation into glutamatergic neurons and cardiomyocytes *in vitro* due to impaired preinitiation complex formation at the promoters of critical differentiation genes. We define an essential role of a core TFIID TAF in differentiation events during mammalian embryogenesis.

## Abstract (web summary)

The role of TFIID core module TAFs (TATA-binding protein-associated factors) in embryogenesis is unknown. Here, the authors show that Taf4 is essential at mid-gestation and for complete neuronal differentiation of embryonic stem cells, but that Taf4a and Taf4b are redundant at early embryonic stages.

## Graphical abstract

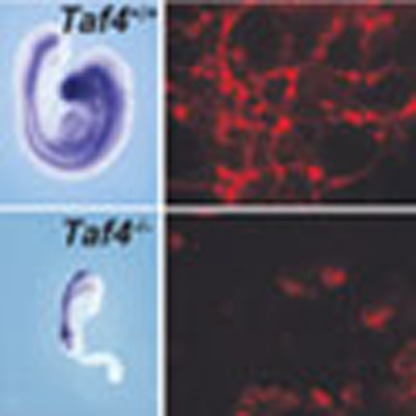

Formation of the RNA polymerase II (Pol II) preinitiation complex (PIC) requires a set of general transcription factors among which is TFIID, a multiprotein complex composed of the TATA-binding protein (TBP) and a set of TBP-associated factors (TAFs)^1^. TFIID assembles from a ‘core complex' comprising TAF4, TAF5, TAF6, TAF9 and TAF12, followed by TAF8-TAF10, and then association with a second module comprising TBP, TAF1, TAF2 and TAF7 (refs 2, 3). In mammals, core TAFs are almost ubiquitously expressed, although cell-specifically expressed paralogues of TBP and a subset of TAFs have been described and their functions defined by mouse knockouts. Taf7l plays a critical role in male germ cell development and in adipocytes^4^,^5^,^6^, Taf4b is essential for male and female fertility^7^,^8^ and Taf9b regulates neuronal gene expression^9^. Trf2 and Trf3, the two TBP paralogues, play essential roles in the male and female germ lines, respectively^10^,^11^.

TAF4 forms a histone fold heterodimer with TAF12 (refs 12, 13, 14) that associates with the TAF6–TAF9 heterodimer and TAF5 to form the TFIID core module. TAF4 is crucial for the structural integrity of the core module and TFIID^1^,^15^. Its paralogue TAF4B also heterodimerizes with TAF12 and integrates into TFIID, thus maintaining TFIID integrity in the absence of TAF4 (ref. 16). While we have used somatic inactivation to address the function of murine Taf4 in mouse embryonic fibroblasts (MEFs), the adult murine epidermis or neonatal liver^16^,^17^,^18^,^19^, its role in embryogenesis is unknown.

Here we characterise *Taf4a*^−/−^ (gene encoding Taf4) embryos showing they survive until E9.5, but exhibit severe growth retardation and specific defects in anterior and ventral patterning and morphogenesis. We also show that *Taf4a*^−/−^ embryonic stem cells (ESCs) are viable, but are unable to properly differentiate into glutamatergic neurons and cardiomyocytes *in vitro* due to impaired PIC formation at the promoters of critical differentiation genes. Thus, while Taf4b can compensate for the absence of Taf4 during the early stages of embryogenesis and in ESCs, Taf4 plays specific roles in differentiation *in vivo* and *in vitro*.

## Results

### Mid-gestation embryonic lethality of *Taf4a* ^−/−^ embryos

Mice with floxed and null *Taf4a* alleles^16^ (*Taf4a*^lox/−^ and *Taf4a*^+/−^ ) were viable, fertile and did not exhibit any apparent abnormalities. Breeding of *Taf4a*^+/−^ mice did not yield any *Taf4a*^−/−^ pups, indicating death *in utero*^16^. We, therefore, analysed embryogenesis at various developmental stages after crosses of *Taf4a*^+/−^ animals.

**Figure 1:**
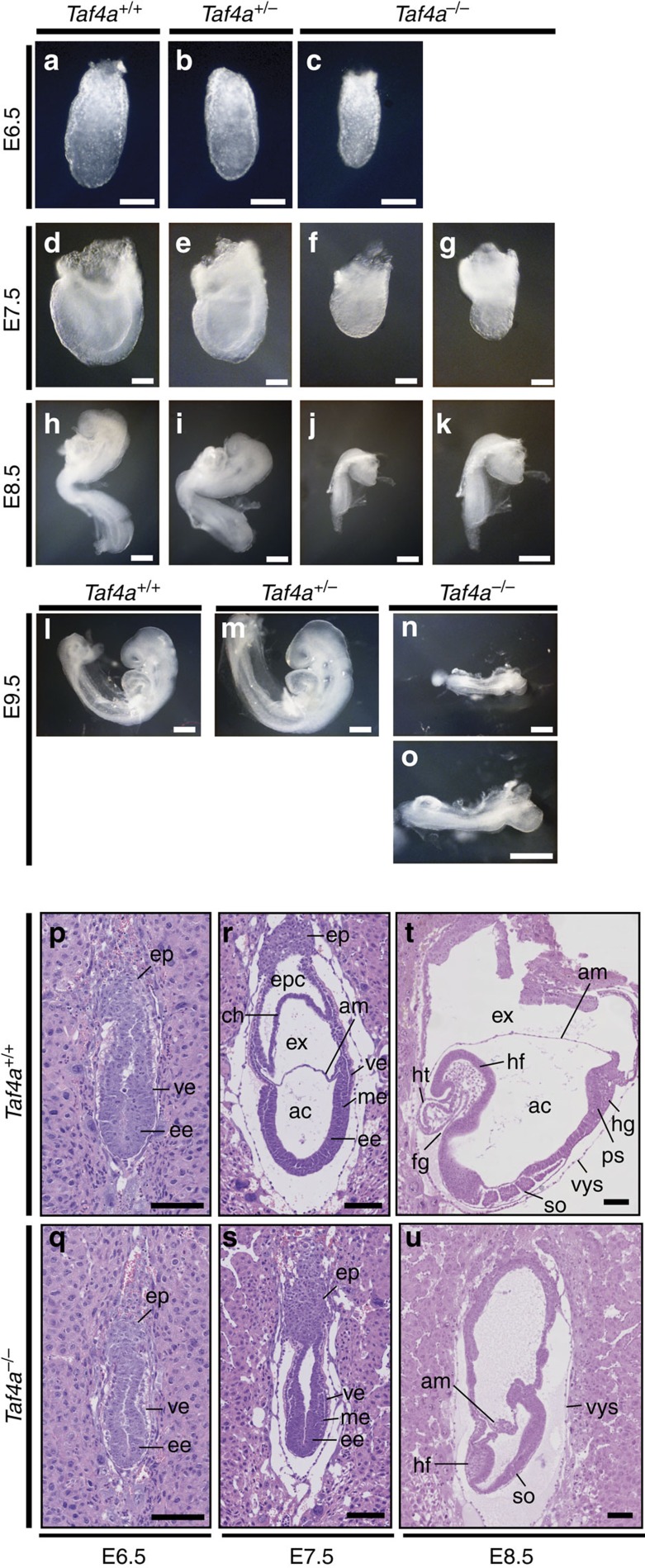
Taf4 is required during mid-gestation. (**a**–**c**) E6.5 stage embryos of the indicated genotypes. (**d**–**g**). E7.5-stage embryos of the indicated genotypes, **g** shows a blow up of **f**. Scale bar, 100 μm. (**h**–**k**). E8.5-stage embryos of the indicated genotypes, **k** shows a blow up of **j**. (**l**–**o**) E9.5-stage embryos of the indicated genotypes, **o** shows a blow up of **n**. Scale bar, 200 μm. (**p**–**u**) Haematoxylin and eosin-stained sections through WT or mutant embryos at the indicated stages. Scale bar, 100 μm. ep, ectoplacental cone; ve, visceral endoderm; ee, embryonic ectoderm; me, mesoderm;, epc, ectoplacental cavity; ch, chorion; ex, exocoelomic cavity; am, amnion; ac, amniotic cavity; hf, headfold; ht, heart; fg, foregut; hg, hindgut; so, somites; vys, visceral yolk sac; ps, primitive streak.

*Taf4a*^−/−^ embryos could be distinguished from *Taf4a*^+/+^ and *Taf4a*^+/−^ embryos by their smaller overall size at E6.5 (Fig. 1a–c). By E7.5 mutant embryos displayed a more compact appearance (Fig. 1d–g), and by E8.5, a shortened body axis and under-developed head and trunk regions (Fig. 1h–k). Between E8.5 and E9.5, mutant embryos showed virtually no increase in overall size, but developmental processes continued with for example formation of a primitive heart. Nevertheless, the overall morphology of *Taf4a*^−/−^ embryos showed that their development did not proceed much beyond that of E8.5 wild-type (WT) embryos (Fig. 1k–m and see below). At E9.5, mutant embryos were severely growth retarded and subsequently resorbed (Fig. 1l–o). Genotyping indicated that *Taf4a*^−/−^ embryos were present in approximately Mendelian ratios until E9.5 after which time they died and were longer detected (Supplementary Table 1).

Histology analysis showed that WT E7.5 embryos had completed division of the proamniotic cavity into the exocoelomic and the amniotic cavities, separated by the amniotic membrane (Fig. 1r), whereas in *Taf4a*^−/−^ embryos this compartmentalization had not occurred resulting in an open proamniotic cavity (Fig. 1s). *Taf4a*^−/−^ embryos showed reduced size and overall resembled WT embryos at the egg cylinder stage at E6.5 (Fig. 1p,q). WT E8.5 embryos underwent major developmental events, including foregut invagination, concurrent headfold and heart tube development, and formation of the first somite pairs (Fig. 1t). In contrast, *Taf4a*^−/−^ embryos displayed severely under-developed anterior structures with no apparent heart structure formation or foregut invagination. Headfold-like mesenchymal tissues were compact, showed poor morphogenesis and reduced size. A large section of the trunk region was missing and somites were small, compact and tightly spaced (Fig. 1u and see below Fig. 3). At E8.5 the developing amniotic structure appeared agglutinated and failed to elongate properly to surround the embryo.

*Taf4a*^−/−^ embryos showed growth defects at E6.5 and most severe by E9.5. To examine this, we measured 5-bromo-2'-deoxyuridine (BrdU) incorporation, but no major change in proliferation rates was seen (Supplementary Fig. 1A). In contrast, we observed increased apoptosis in mutant embryos assessed by terminal deoxynucleotidyl nick-end labelling on whole-mount embryos and paraffin sections (Supplementary Fig. 1B,C). Increased apoptosis occurring as early as E7.5, rather than reduced cell proliferation, therefore contributes to the smaller size of the *Taf4a*^−/−^ embryos.

### Overlapping embryonic expression of *Taf4a* and *Taf4b*

Whole-mount *in situ* hybridization indicated ubiquitous expression of *Taf4a* (Supplementary Fig. 2A). A specific signal was observed with the anti-sense probe throughout the embryo and the extraembryonic region at E6.5 and E7.5. By E8.5, *Taf4a* mRNA was detected in the entire embryo and in the yolk sac. Consistent with the *in situ* hybridization, Taf4 protein was detected throughout the E7.5 WT embryo, but was absent from the mutant embryos (Supplementary Fig. 2B).

As *Taf4a*^−/−^ embryos die much later than germ line knockouts for Tbp or other core Tafs, it is possible that Taf4b may at least partially compensate for Taf4 loss. *In situ* hybridization showed ubiquitous *Taf4b* expression at E6.5 that was more pronounced in the extraembryonic region (Supplementary Fig. 2A). At E7.5 *Taf4b* was expressed throughout the epiblast and extraembryonic regions. Notably, however, expression in the visceral endoderm and the definitive endodermal layer was weaker than *Taf4a*. Distinct enrichment in *Taf4b* expression in regions corresponding to the ectoplacental cavity and in a ring of extraembryonic ectoderm at the mid proximo-distal domain was observed. At E8.5 *Taf4b* expression closely resembled that of *Taf4a*, with the exception of the yolk sac where staining was weaker than *Taf4a*.

The overlapping expression suggests *Taf4b* may partially compensate for lack of *Taf4a* and thus account for the later death of *Taf4a* mutants. Indeed, *Taf4b* expression was detected throughout the *Taf4a* mutant embryos at E8.5 (Supplementary Fig. 2C). Quantitative reverse transcription–PCR (RTq–PCR) analysis confirmed that its expression was virtually unchanged in the mutant embryos (Supplementary Fig. 2D). All other TFIID Tafs were comparably expressed in WT and mutant embryos. Only *Taf5*, *Taf6* and *Taf13* showed >2-fold increased expression in the mutant embryos (Supplementary Fig. 2D).

### Defective heart formation in *Taf4a* ^−/−^ embryos

**Figure 2:**
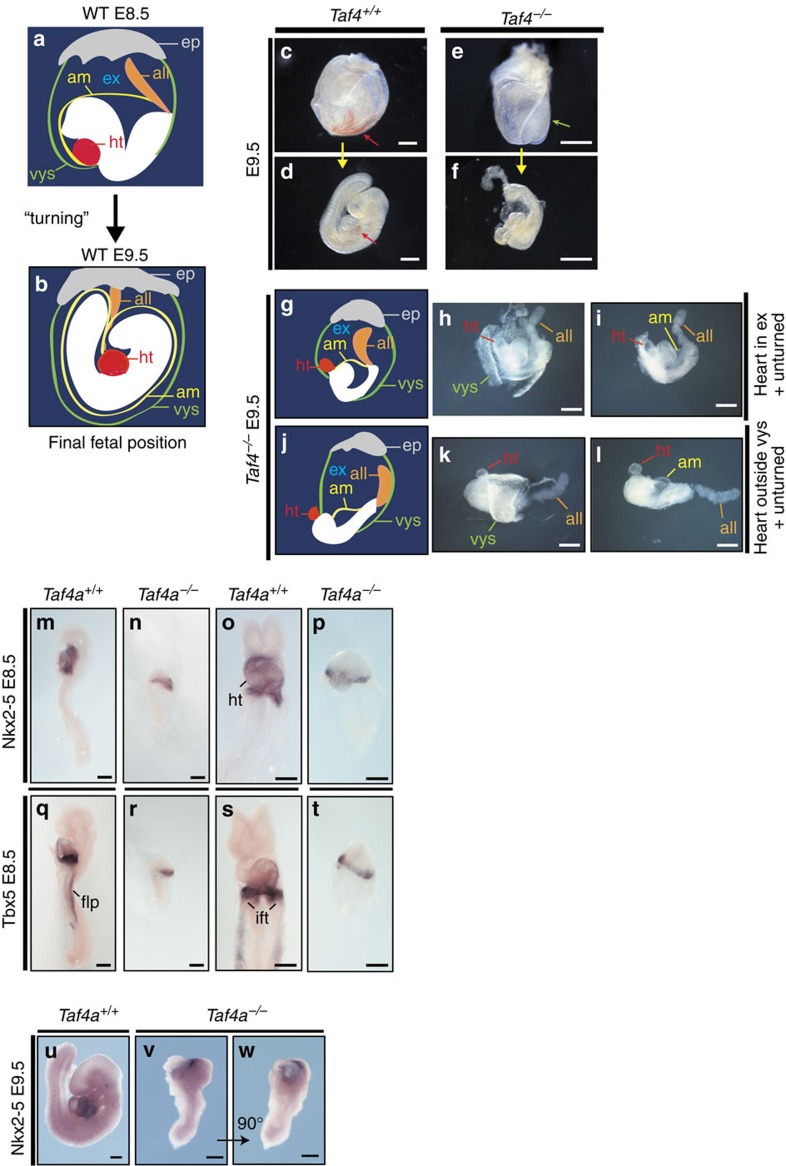
Taf4 is required for correct positioning of head and heart. (**a**,**b**). Schematic representations illustrate normal development at E8.5 and E9.5 highlighting positions of head and heart during embryo turning. (**c**–**f**) WT and mutant embryos at E9.5 before and after dissection from the yolk sac highlighting the blood-filled yolk sac vascular plexus in control embryos (red arrow in **c**) and the embryo protruding partially out of the yolk sac (green arrow in **e**). WT embryos show a strong accumulation of blood in the atrium of the heart (red arrow in **d**), which was absent in *Taf4a*^−/−^ embryos (**f**). (**g**–**i**) Schematic illustration of observed mutant phenotypes at E9.5. All mutant embryos fail to undergo turning at E9.5 and fusion of the allantois to the chorion. In addition to this, mutant embryos develop heart structures either in the exocoelomic cavity (**h**–**i**) or outside of the visceral yolk sac (**k**–**l**). (**m**–**w**) *In situ* hybridization of wild-type and *Taf4a*^−/−^ embryos for the cardiac transcription factors *Nkx2-5* at E8.5 (**m**–**p**) or *Tbx5* at E8.5 (**q**–**t**) and E9.5 (**u**–**w**). ep, ectoplacental cone; all, allantois; ht, heart; flp, forelimb precursors; ift, inflow tract. Scale bar, 500 μm (**c**–**f**), 300 μm (**h**-**i**), 200 μm (**m**–**w**).

The yolk sac comprises a bilayer structure of mesoderm- and endoderm-derived cells crucial for embryo survival. The first blood cells and blood islands develop in the mesodermal compartment of the yolk sac^20^,^21^,^22^. Before E8.5 mouse embryos are in a lordotic position such that the embryo initially faces the outside of the egg cylinder (ref. 23 and Fig. 2a). Around E8.5 the embryo rotates around its own axis placing the heart in a ventral position (Fig. 2a,b) and the amnion enlarges to surround the whole embryo. The exocoelome lining consisting of visceral endoderm and mesoderm also expands and eventually surrounds the whole embryo as the visceral yolk sac.

*Taf4a*^−/−^ embryos failed to turn by E9.5 developing partially outside the yolk sac that was reduced compared with WT (Fig. 2c–f). Mutant embryos lacked the blood-filled vascular plexus and the developing blood vessels seen in WT yolk sacks, suggesting defective erythroid cell generation (Fig. 2c–f). In mutant yolk sacks, endothelial cells were present, but did not form organized vascular structures (Supplementary Fig. 3A). Immunolabeling with PECAM-1, an endothelial cell-specific maker, revealed formation of a primitive vascular system only on the dorsal surface of the *Taf4a*^−/−^ embryos adjacent to the neural tube that failed to pervade the interior of the embryo (Supplementary Fig. 3A). Defective allantois fusion to the chorionic plate was also observed. At E9.5, fusion is complete in 100% of the WT embryos, compared with only 10% of mutant embryos (Fig. 2k,l and Table 1).

E9.5 WT embryos displayed a well-organized heart tube with erythrocytes visible in the inflow region (Fig. 2d), whereas mutant embryos showed primitive cardiac structures bulging from the headfold base often developing outside the amnion in the exocoelomic cavity (Fig. 2g–i and Table 1). In rare cases, heart structures developed outside the visceral yolk sac (Fig. 2j–l). While 100% of WT embryos developed a primitive heart tube by E8.5, only 8% of mutant embryos showed a small bulge indicating a severely retarded initiation of heart tube formation (Fig. 2g–l and Table 1). At E9.5 all WT hearts showed contractions on dissection, whereas this was never observed in mutant embryos.

*In situ* hybridization showed specfic expression of cardiac transcription factor Nkx2–5 in the prospective myocardium of the atria, ventricles, and outflow tract in E8.5 WT embryos (Fig. 2m,o). In contrast, mutant embryos exhibited a crescent-like expression domain with no evident heart chamber formation indicating that although cardiac lineage specification occured, heart tube morphogenesis was defective (Fig. 2n,p). Expression of a second cardiac transcription factor Tbx5 was similar to Nkx2–5 although this factor was expressed at higher levels in inflow tract structures and showed additional staining in the prospective forelimb field (Fig. 2q,s). In mutant embryos, Tbx5 showed a crescent-like expression domain, with no expression in the prospective forelimb (Fig. 2r,t). These crescent-like domains in the E8.5 mutant embryos closely resembled the expression pattern of heart-specific markers in the cardiac crescent of E7.5 WT embryos. Importantly however, histology analyses and *in situ* hybridization indicated mis-localization of the primitive heart structures anterior to the headfolds rather than in the ventral position in keeping with the absence of turning of the mutant embryos. This was highlighted by *in situ* hybridization with Nkx2–5 at E9.5 labelling the developing heart structure at the anterior pole of the embryo next to the headfolds rather than in a ventral position as in WT (Fig. 2u–w). *Taf4a*^−/−^ embryos therefore display defective cardiac development that, together with lack of chorio-allantoic fusion and defective yolk sac and intra-embryonic vascularization, likely accounts for embryonic death at mid-gestation.

### Abnormal anterior patterning of *Taf4a* ^−/−^ embryos

**Figure 3:**
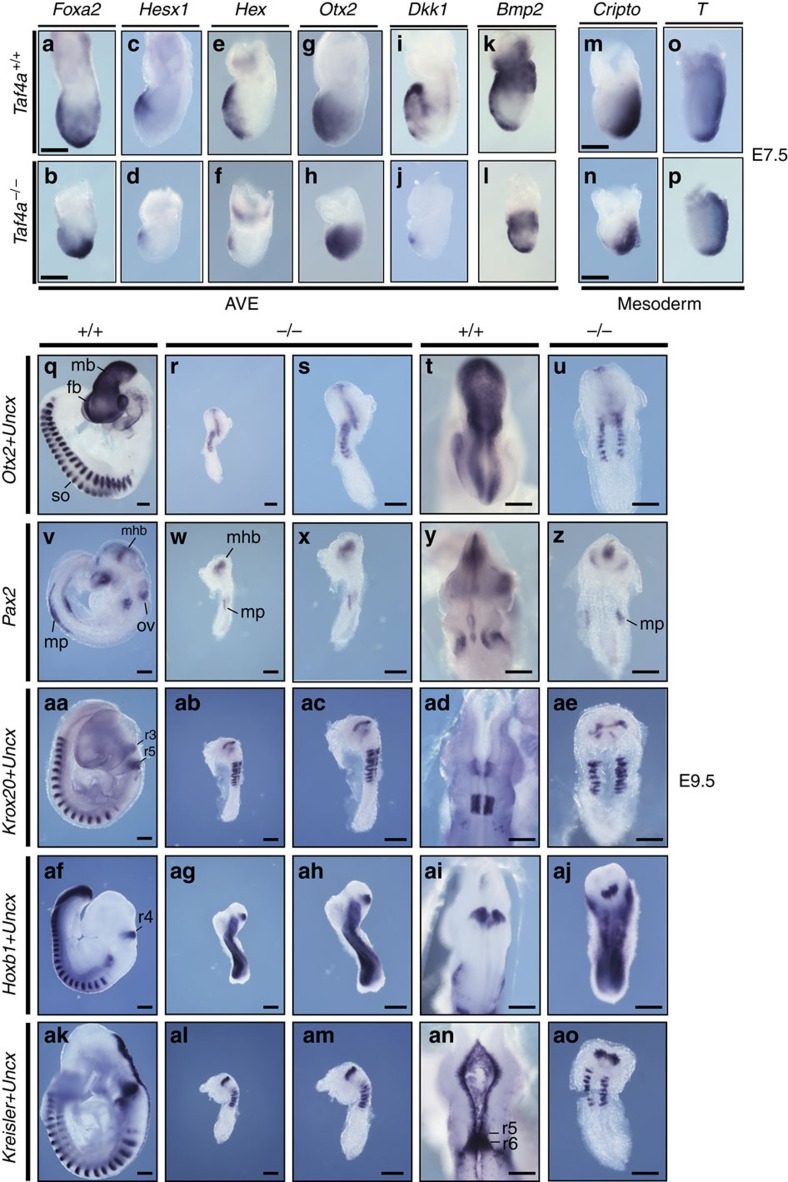
Abnormal patterning of *Taf4a*^−/−^ embryos. (**a**–**o**) (upper row). *In situ* hybridization with the indicated probes for anterior visceral endoderm (AVE) and mesoderm markers on E7.5 WT embryos. (**b**–**p**) (lower row). *In situ* hybridization with the indicated probes on E7.5 *Taf4*^−/−^ embryos. (**q**–**ao**). *In situ* hybridization with neuronal markers in E9.5 WT or *Taf4*^−/−^ embryos (lateral views (**q**–**ak**); ventral view R dorsal views T-AN) and (lateral views RP-AL, higher magnifications of same embryos **s**–**am**; ventral view U; dorsal views **z**–**ao**). Scale bar, 200 μm in all panels.

We next used specific markers to investigate defective anterior development of *Taf4a*^−/−^ embryos, The anterior visceral endoderm (AVE) regulates initial steps of head, heart and foregut positioning. Defective expression of AVE markers leads to severe anterior truncation phenotypes^24^. Expression domains of several AVE markers in E7.5 *Taf4a*^−/−^ embryos were either reduced (*Hesx1*, *Hex* and *Dkk*1) or showed retarded formation (*Foxa2*, *Otx2*, Fig. 3a–l). In contrast, expression domains of mesodermal markers *Brachyury* (*T*) and *Cripto* were comparable in WT and mutant embryos (Fig. 3m–p).

To investigate differentiation of specific anterior structures, we studied expression of forebrain, midbrain and hindbrain markers. *Otx2* is required for induction and maintenance of the anterior neural plate^25^,^26^ and specification of forebrain and midbrain regions. The *Otx2* expression domain was diminished in E9.5 *Taf4a*^−/−^ mutants, particularly the forebrain was strongly reduced (Fig. 3q–u). *Pax2* was expressed in the developing midbrain–hindbrain region, the otic vesicle, and the mesonephros in WT embryos, whereas in mutant embryos midbrain–hindbrain staining was reduced, and the presence of nephrogenic precursor cells was observed. In contrast, no staining of presumptive otic vesicle structures was observed (Fig. 3v–z).

In WT E9.5 embryos, *Krox20* marked rhombomeres 3 and 5. In mutant embryos, labelled structures were thinner and disorganized with rhombomere 5 more affected than rhombomere 3 (Fig. 3aa–ae). *Hoxb1*, a marker for rhombomere 4, was clearly detected in mutant embryos (Fig. 3af–aj), whereas *Mafb* (*Kreisler)*, expressed in rhombomeres 5 and 6 in WT embryos (Fig. 3ak,an), stained only a single stripe in *Taf4a*^−/−^ mutants (Fig. 3al,am,ao), which in some instances, was thinner than the *Hoxb1*-positive stripe (compare Fig. 3ag,al). Thus, although the anterior region of mutant embryos was severely atrophied, this did not reflect a complete lack of forebrain/midbrain/hindbrain specification, all of which were present albeit both reduced in size and disorganized. *Uncx* staining showed fewer (typically 8–11) and irregularly shaped somites in E9.5 *Taf4a*^−/−^ mutants.

The above analyses revealed an immediate proximity of the hindbrain and somite markers (*Uncx*) and a reduction or absence of the hindbrain posterior to rhombomere 5 along with a significant portion of the trunk. We analysed the anterior–posterior axis and gut development in mutant embryos. *Foxa2* and *Shh* labelled the notochord and developing hindgut in E9.5 WT embryos, but only one structure in mutant embryos (Supplementary Fig. 3B). Brachyury (*T*) staining indicated the labelled structure was the notochord showing interruption at one or several places, perhaps due to local apoptosis or tissue distortion (Supplementary Fig. 3C). The notochord showed stronger anterior staining by *Brachyury* and *Foxa2* indicating defective elongation along the anterior–posterior axis. Nevertheless, mutant embryos maintained strong *Brachyury* staining in their posterior region, which was proportionally larger than in WT embryos at E8.5 and E9.5. This could indicate a defect in specification and differentiation of axial mesendodermal structures. *Taf4a*^−/−^ embryos therefore fail to develop a detectable gut tube consistent with histology analysis that showed lack of a foregut pocket E8.5 (Fig. 1u) and their shortened notochord is consistent with the absence of an elongated trunk region.

### Reduced neural crest domain in *Taf4a* ^−/−^ embryos

Having defined defective anterior and trunk development, we next investigated neural crest cells (NCCs) normally induced at the midbrain level and as a wave extending towards the tail region. Defects in NCCs may contribute to the defects observed in the cephalic and trunk region of the *Taf4a*^−/−^ embryos.

**Figure 4:**
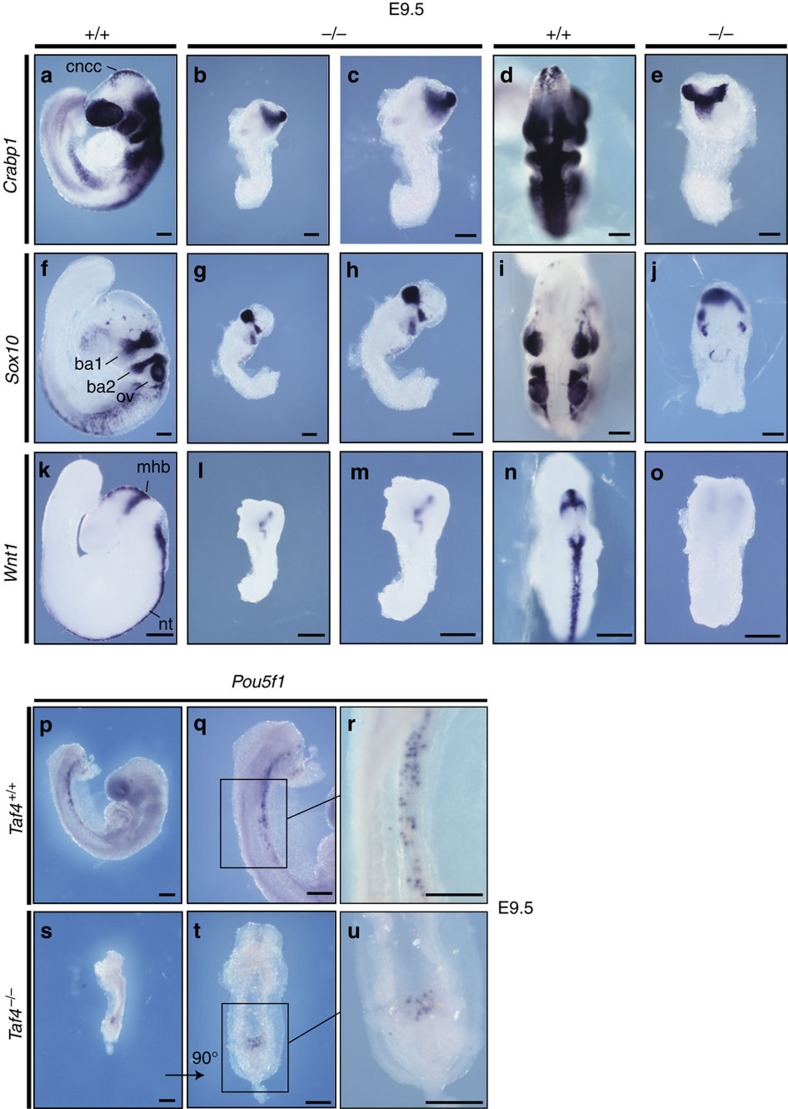
Neural crest cell markers and primordial germ cells in *Taf4* mutant embryos. (**a**–**o**) *In situ* hybridizations for neural crest markers *Sox10*, *Crabp1* and *Wnt1* in E9.5 control (lateral views **a**,**f**,**k** dorsal views **d**,**i**,**n**) and *Taf4a*^−/−^ embryos (lateral views **b**,**g**,**l** higher magnifications of the same embryos **c**,**h**,**m** dorsal views E, J, O ). (**p**–**u**). *In situ* hybridizations at E9.5 with *Pou5f1* (*Oct4*) as a marker for primordial germ cells (PGCs). ba1, first branchial arch; ba2, second branchial arch; ov, otic vesicle; cncc, cranial neural crest cells; mhb, midbrain–hindbrain boundary; nt, neural tube. Scale bar, 200 μm in all panels.

In E9.5 WT embryos, *Crabp1* labelled the neural tube and NCCs originating in the posterior hindbrain and anterior trunk regions that give rise to cranial NCCs migrating into the branchial arches and posterior regions (Fig. 4a,d). A severely reduced *Crabp1* expression domain was seen in mutant embryos, indicating they were completely devoid of migrating NCCs in both the cranial and posterior regions (Fig. 4b,c,e). The residual y-shaped expression domain may correspond to part of the neural tube and primitive branchial arch 1. Similarly, *Sox10*, a marker with more restricted expression domains in the otic vesicle and the NCCs migrating into the branchial arches (Fig. 4f,i), also showed severely reduced expression in the mutant embryo and no migrating NCCs were observed (Fig. 4g,h,j). Furthermore, *Wnt1* expression was absent from the spinal cord and NCCs (Fig. 4k–o). *Taf4a*^−/−^ embryos therefore lack a large portion of the NCCs migrating towards craniofacial structures and more posterior trunk regions.

### Taf4 is dispensible for primordial germ cell generation

Given the role of Taf4 in reprogramming (ref. 27 and see below), we specifically investigated the presence of primordial germ cells (PGCs) in *Taf4a*^−/−^ embryos. PGCs colonize the developing gonads by migration giving rise to oocytes and spermatocytes in adults^28^. PGCs can be identified at around E7.25 (refs 29, 30) and begin to migrate through the primitive streak to the developing hindgut, and by ≈E9.5 towards the genital ridges. Mutant embryos lack a properly developed hindgut mesentery at E9.5. We investigated whether mutants were able to initiate PGC development. At E7.5 *Pou5f1* (*Oct4)* is exclusively expressed by the newly formed PGCs, and expression persists as they migrate and proliferate. We readily detected *Pou5f1*-expressing PGCs in the WT hindgut mesentery at E9.5 (Fig. 4p–r). In mutant embryos, *Pou5f1*-expressing PGCs could be observed, but were around 10-fold less numerous than in WT (Fig. 4s–u). These PGCs accumulated at the presumptive entry site into the hindgut lacking in mutant embryos. Thus, PCG specification takes place in absence of Taf4, but they cannot migrate and pursue their proliferation due to the absence of the hindgut.

### *Taf4a* ^−/−^ ESCs are viable and comprise TFIID containing Taf4b

The lethality, reduced size and pleiotropic defects of the *Taf4a*^−/−^ embryos preclude biochemical and functional analysis of specific differentiation events. Nevertheless, as death occured at mid-gestation, it should be possible to derive *Taf4a*^−/−^ ESCs and use them to address the role of Taf4 in specific differentiation programmes. We isolated ESCs from blastocysts of *Taf4a*^+/−^ heterozygous crosses and established two homozygous null lines and two WT lines from these crosses. The absence of Taf4 protein in *Taf4a*^−/−^ ESCs was confirmed by immunostaining, while pluripotency markers Pou5f1, Nanog and Sox2 were normally expressed (Supplementary Fig. 4A–C). WT and *Taf4a*^−/−^ ESCs showed comparable alkaline phosphatase staining, proliferation rates and cell cycle profiles (Supplementary Fig 4D–F). Comparative RNA sequencing (RNA-seq) revealed only minor differences in gene expression. Around 40 genes were upregulated although only 12 showed more than a threefold change, whereas 166 genes were downregulated (Supplementary Fig. 4G) including Noggin and several genes associated with Wnt signalling. However, their altered expression did not elicit detectable changes in cell physiology and no change in pluripotency gene expression was observed. Together these data show that *Taf4a*^−/−^ ESCs are viable and comparable in most essential parameters to WT ESCs.

**Figure 5:**
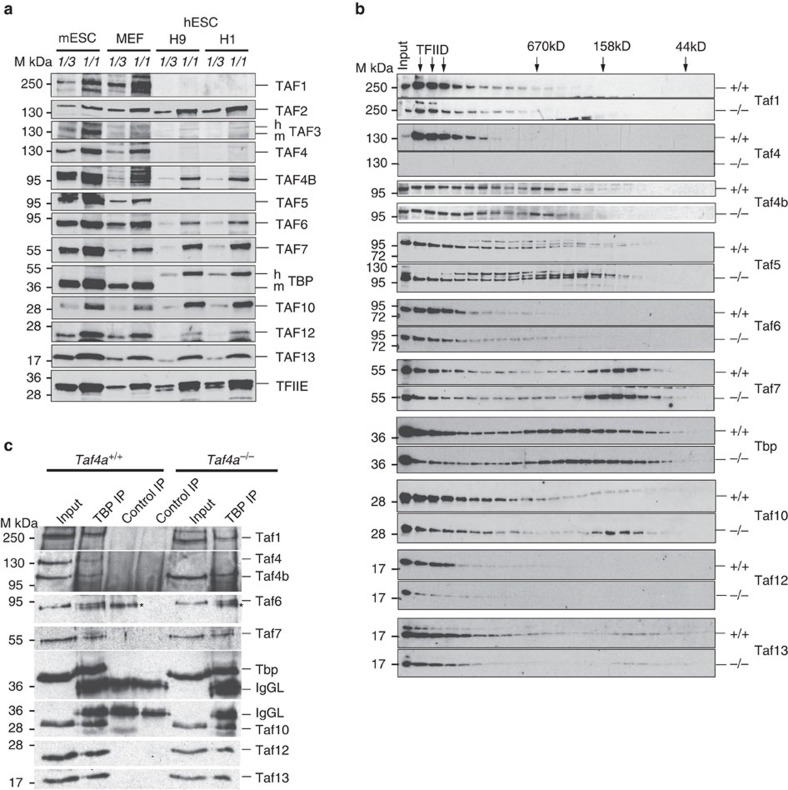
Biochemical analysis of TFIID in *Taf4a*^−/−^ ES cells. (**a**). Immunoblots showing expression of TFIID subunits in ES cells, MEFs and two human ES cell lines, H1 and H9. (**b**). Immmunoblots on gel filtration fractions of nuclear extracts from WT and mutant ES cells. (**c**). Immunoblots of Tbp IPs from the pooled TFIID fractions indicated by arrows in **b**. * indicates a non-specific band migrating just below Taf6.

To characterise ESC TFIID and to compare it with human ESCs and MEFs, we performed western blot analysis. WT mouse ESCs express Tbp and all tested Tafs, including Taf4b. For equivalent amounts of protein extract, expression of many Tafs was higher in ESCs than in MEFs (Fig. 5a), consistent with previous reports^27^. In two human ESC lines, no expression of TAF1, TAF4 and TAF5 was seen, although TAF4b was detected. These observations confirm previous reports of non-canonical TFIID composition in human ESCs^31^, and highlight fundamental differences in the biochemistry of mouse and human ESCs.

Comparison of WT and *Taf4a*^−/−^ ESCs indicated comparable expression of most TAFs. Tbp was immunoprecipitated (IP) from both lines and equivalent amounts of Tbp loaded on immunoblots (Supplementary Fig. 5). Taf4b was co-precipitated with Tbp in both the presence and absence of Taf4 indicating that Taf4b maintained TFIID integrity in *Taf4a*^−/−^ ESCs. Nevertheless, reduced levels of several TAFs, with respect to Tbp, were observed in the Tbp IP from the *Taf4a*^−/−^ ESCs suggesting that Taf4b expression was not sufficient to maintain an overall TFIID level comparable to WT ESCs.

We then performed gel filtration and analysed the presence of Tbp and TAFs in the high-molecular mass fractions. Tbp and TAFs coeluted in a high molecular mass complex of around 2 mDa in WT extracts (Fig. 5b). Taf4b was also present in these fractions, although a subfraction eluted in a lower molecular mass complex. In *Taf4a*^−/−^ extracts, Taf4b was present along with Tbp and other tested TAFs in high-molecular mass fractions, although Tbp and many TAFs were present in reduced amounts in particular Taf7 and Taf10 that were increased in lower molecular weight fractions.

To investigate whether the high-molecular mass fractions correspond to TFIID, the indicated fractions were pooled and subjected to Tbp IP. In fractions from WT and mutant ESCs, Tafs 1, 4b, 6, 7, 10, 12 and 13 were co-precipitated with Tbp showing formation of a TFIID complex in both cell types (Fig. 5c). In fractions from WT cells, both Taf4 and Taf4b were precipitated, while in the fractions from mutant cells only Taf4b was present. Taf4b therefore integrates in TFIID in *Taf4a*^−/−^ ESCs, but these cells contain lower overall levels of TFIID as the presence of Taf4b cannot quantitatively compensate Taf4 loss.

To further verify potential redundancy between Taf4 and Taf4b, we transfected WT and *Taf4a*^−/−^ ESCs with control small interfering RNA (siRNA) and siRNAs directed against Taf4b. After control siRNA and siTaf4b silencing in WT ESCs and replating the transfected cells, comparable numbers of alkaline phosphatase staining colonies were observed expressing comparable levels of Nanog, showing that Taf4b knockdown did not compromise cell viability (Supplementary Fig. 6A–C). In contrast, Taf4b silencing in *Taf4a*^−/−^ ESCs led to a strong reduction in the number of colonies and surviving cells showed normal Taf4b and Nanog expression. Hence, Taf4b silencing in *Taf4a*^−/−^ ESCs strongly compromised cell viability and only cells that had escaped transfection formed colonies. These results indicate that ESCs lacking either Taf4 of Taf4b were viable. In contrast, loss of both proteins through genetic deletion of Taf4 and subsequent Taf4b silencing resulted in a loss of viability. Taf4 and Taf4b are therefore redundant in terms of assuring cell ESC viability.

### Defective neuronal differentiation of *Taf4a* ^−/−^ ES cells

*Taf4a*^−/−^ ESCs were grown as embryoid bodies (EBs) to determine their spontaneous differentiation capacity over a period of 14 days. *Taf4a*^−/−^ EBs initially appeared indistinguishable from WT EBs, but at later stages, gave rise to fewer cystic-like structures (Supplementary Fig. 7A) that resemble the developing yolk sac *in vivo*^32^. Hence, *in vitro* differentiated *Taf4a*^−/−^ ES cells recapitulated to some extent the phenotype of the *Taf4a*^−/−^ embryos. *Taf4* was expressed throughout WT EB differentiation, while *Taf4b* expression was strongly decreased, but *Taf4b* was maintained in the mutant cells (Supplementary Fig. 7B).

We investigated whether *Taf4a*^−/−^ ESCs could give rise to the three primary germ layers. Expression of the pluripotency markers *Nanog* and *Dppa3* was reduced, but incompletely repressed compared with WT (Supplementary Fig. 7B). The early endoderm and mesoderm markers *Gata4* and *Gata6* were induced, whereas late endodermal lineage markers like *Foxa2*, and *Afp* were strongly downregulated (Supplementary Fig. 7B). Mesendodermal markers Brachyury (*T*) and Goosecoid (*Gsc*) showed a strong upregulation in day 6 EBs before their expression returned to normal levels, indicating that Taf4 may regulate the fine-tuning of their expression. Expression of the early cardiac marker *Nkx2–5* and its downstream target gene *Anf* was also reduced. *Taf4a*^−/−^ ESCs can therefore initiate differentiation into early mesoendodermal-type cells, whereas later stage differentiation markers were more strongly affected.

Expression of the early ectodermal marker *Fgf5* was induced, but expression of later neuronal markers such as *Pax6* and *Mtap2* and *Dcx* was strongly reduced (Supplementary Fig. 7B). To investigate the role of Taf4 in neuronal differentiation, we differentiated WT and *Taf4a*^−/−^ ESCs into glutamatergic neurons by treating EBs with all-*trans* retinoic acid (RA) (Supplementary Fig. 8A, refs 33, 34). Expression of the vesicular glutamate transporter Vglut2 as well as Taf4 was observed in such neurons cultured for 12 days (Supplementary Fig. 8B).

**Figure 6:**
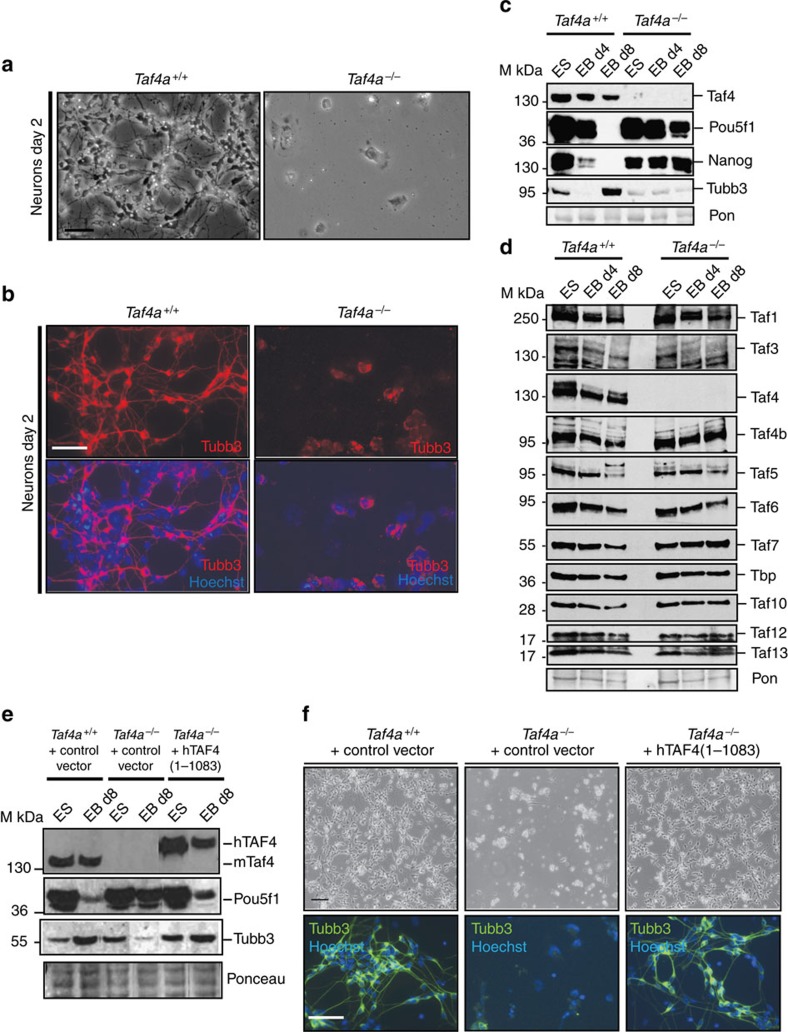
Taf4 is required for RA-driven neuronal ES cell differentiation. (**a**) Phase-contrast microscopy of RA-treated EBs differentiated into neurons 2 days after plating. (**b**) Tubb3 labelling of RA-treated EBs differentiated into neurons 2 days after plating. (**c**,**d**). Immunoblots on extracts from differenting ES cells and EBs with the indicated antibodies. The membranes were stained with Ponceau as loading controls. (**e**). Immunoblots on extracts from ES cell lines engineered to re-express recombinant Taf4. (**f**). Phase-contrast microscopy and Tubb3 labelling of RA-treated EBs differentiated into neurons 2 days after plating. All images were taken at × 20 magnification. Scale bars 100 μm.

Dissociated RA-treated WT EBs differentiated into neuronal precursors shortly after plating and adopted neuronal morphology with abundant axons and prominent Tubb3 staining after 2 days (Fig. 6a,b). In contrast, mutant EBs failed to generate neuronal precursors with an axonal network and most cells died within a day. After 1–2 days, the few remaining cells appeared flattened and enlarged. Western blot showed only weak Tubb3 expression in mutant cells, but also revealed defective repression of Pou5f1 and Nanog whose expression was strongly repressed after differentiation of WT EBs (Fig. 6c). Taf4 is therefore essential for RA-induced neuronal differentiation and for pluripotency gene repression.

Immunoblot analysis showed a mild overall reduction in Tbp and Taf expression by day 8 of WT EB differentiation (Fig. 6d). On differentiation of mutant EBs, the overall reduction in Taf levels was less marked with no decreased Taf4b expression, whereas Taf3 and Taf5 were reduced.

To demonstrate that defective differentiation was due to Taf4 loss, we generated *Taf4a*^−/−^ ESCs stably re-expressing exogenous full-length Taf4 (Fig. 6e). WT ESCs isolated in parallel after transfection with control vector showed normal neuronal differentiation with an abundant Tubb3-stained axonal network, whereas the *Taf4a*^−/−^ EBs transfected with control vector showed no neuronal differentiation (Fig. 6f). Importantly, the rescue *Taf4a*^−/−^ line showed comparable differentiation to the WT lines. Defective differentiation was therefore directly attributable to Taf4 loss and restored by its re-expression. We also attempted to rescue differentiation by the expression of Taf4 (372–1,083) and Taf4 (805–1,083); however, no stable clones could be isolated suggesting that expression of truncated Taf4 was toxic in these cells. The integrity of Taf4 is therefore required to rescue the differentiation defects.

**Figure 7:**
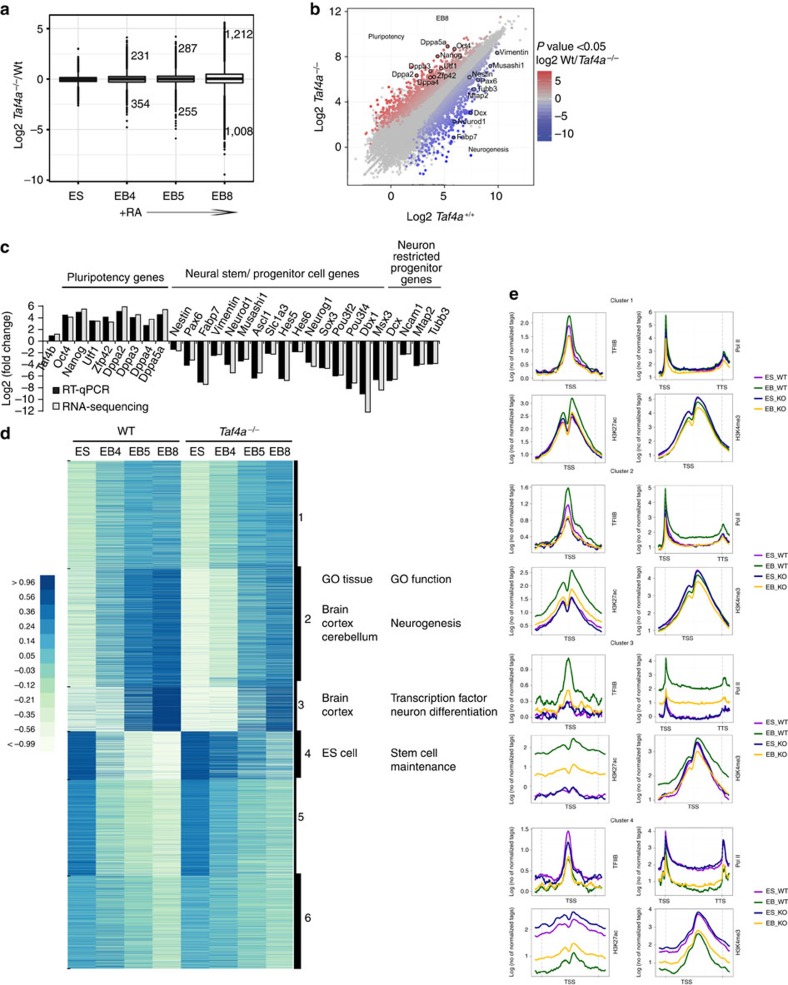
Taf4 is required for activation of neurogenic genes in differentiating ES cells. (**a**). Gene expression changes at different stages of differentiation in *Taf4a*^−/−^ compared with WT ES cells. The numbers of genes with altered expression and their fold changes at each stage are indicated. (**b**). Global comparison of RNA-seq data in *Taf4a*^−/−^ compared to WT at the EB8 stage. The expression of several neurogenic and pluripotency genes are highlighted. (**c**). Comparison of the changes in expression of selected marker genes measured by RNA-seq and by RT–qPCR. (**d**). Kinetics of gene expression and their alterations during differentiation are represented as a clustered heatmap. The ontology terms associated with the genes in clusters 2–4 that are most affected by Taf4 loss are indicated. (**e**). Metaprofiles for the indicated ChIP-seq data sets corresponding to genes in clusters 2–4 are shown. For TFIIB, H3K4me3 and H3K27ac the profiles are centred on the TSS. For Pol II the profiles are show over the gene from the transcription start site (TSS) to the transcription termination site (TTS).

We performed RNA-seq on two independent WT and mutant ESC lines before (EB4) and 24 (EB5) and 96 h (EB8) after RA treatment. At EB4 585 deregulated genes were observed (354 down and 231 up, Fig. 7a). Similarly, at EB5, 255 upregulated and 287 downregulated genes were detected, but the highest number of deregulated genes was observed at EB8 (1,212 up and 1,008 down). Thus, Taf4b can replace Taf4 to maintain cellular housekeeping functions and pluripotency and to a limited extent the initial stages of differentiation, but Taf4 is required for full differentiation with progressively more severe alterations in gene expression as differentiation progressed (Fig. 7a).

At EB8, genes involved in neurogenesis are strongly downregulated, whereas many pluripotency genes are prominent in the upregulated class (Fig. 7b,c). Downregulated genes are highly associated with the brain, while upregulated genes are associated with ESCs and spermatogenesis reflecting the presence of stem-like genes expressed in PGCs and spermatogonia (Supplementary Fig. 8C). Clustering identified genes with distinct kinetics of expression in WT cells and altered expression in *Taf4a*^−/−^ cells. Three clusters show the most prominent changes. Genes in cluster 2 were upregulated when WT ES cells were grown as EBs, but were most strongly induced after RA treatment at EB5 and were highly enriched in brain and neurogenesis functions (Fig. 7d). In mutant ESCs, their activation was strongly reduced whereby expression at EB8 was comparable to that of WT EB4. Genes in cluster 3 were also strongly enriched in brain and neurogenesis, but were mainly activated between EB5 and EB8 and were strongly reduced in mutant cells. In contrast, genes in cluster 4 are ES-expressed and pluripotency genes downregulated in WT EBs, then switched off during their differentiation, whereas in *Taf4a*^−/−^ EBs, their expression persisted during differentiation.

Similar to *Taf4a*^−/−^ cells *in vitro*, expression of several neuronal genes was downregulated in *Taf4a*^−/−^ embryos showing that Taf4 is also required for their normal expression *in vivo* (Supplementary Fig. 8D). Expression of *Pou5f1*, and *Nanog* was, however, repressed in *Taf4a*^−/−^ embryos, but that of other pluripotency associated genes like *Zfp42* and *Dppa5a* remained elevated at E9.5.

The above data show that WT ESCs differentiated into neuronal precursors that switched off pluripotency gene expression, whereas *Taf4a*^−/−^ ESCs adopt a unique state with partially induced expression of differentiation genes, and partially repressed expression of pluripotency genes.

### Defective PIC formation at neuronal genes

The above classification based on kinetics of gene expression also reflects different regulatory mechanisms as revealed by ChIP-seq. TFIIB, Pol II, H3K4me3 and H3K27ac were present at promoters of genes in cluster 1 in WT ESCs. Consistent with increased expression of these genes during differentiation, TFIIB, Pol II and H3K27ac were increased at EB8 (Fig. 7e). In contrast, lower occupancy was seen in *Taf4a*^−/−^ ESCs and little change was observed at EB8 in accordance with their reduced activation. TFIIB, Pol II, H3K4me3 and low levels of H3K27ac were observed at cluster 2 promoters in WT and mutant ESCs and activation of these genes was associated with increased occupancy in WT EB8, but not in *Taf4a*^−/−^ EB8. Increased elongating Pol II was also observed in WT, but not mutant EB8. The reduced activation of these genes therefore results from diminished PIC formation and elongating Pol II.

In contrast to clusters 1 and 2, where basal levels of PIC formation and Pol II occupancy were observed in ESCs, later stage activation of cluster 3 genes was associated with high levels of *de novo* TFIIB and Pol II recruitment and a potent increase in H3K27ac in WT EB8, but not mutant EB8 (Fig. 7e). Nevertheless, H3K4me3 was observed at these promoters in both WT and mutant ESCs suggesting that even in the absence of PIC formation they were epigenetically marked for future activation. Downregulation of genes in cluster 4 during differentiation was associated with strongly diminished TFIIB, Pol II, H3K27ac and H3K4me3 in WT EB8 showing diminshed PIC formation that was less marked in mutant cells. Consequently, Tbp was redistributed during differentiation of WT ESCs with around 14,000 sites showing preferential or unique occupancy at EB8, while occupancy of many ES sites was strongly reduced (Supplementary Fig. 9A). Tbp was enriched at the TSS of 2,086 genes mainly present in clusters 2 and 3 and strongly enriched in ontology terms associated with brain and the nervous system consistent with redistribution of Tbp from ES-expressed/pluripotency genes to those involved in neuronal differentiation (Supplementary Fig. 9B).

In contrast, in *Taf4a*^−/−^ EB8, little occupancy of the neuronal associated WT EB8 sites was observed (Supplementary Fig. 9A). Instead, Tbp was enriched at EB8 at around 3,000 genes associated with embryos and a variety of tissues from the different germ layers (Supplementary Fig. 9C). A smaller group of around 3,000 sites appeared more strongly occupied in mutant EB8 that were associated with 400 genes with only poorly enriched ontology scores.

Together these observations are consistent with the idea that Tbp was redistributed from ES-expressed/pluripotency to neurogenic genes during differentiation of WT ESCs, whereas in *Taf4a*^−/−^ ESCs, Tbp redistribution was diminished, and it remained associated with ES-expressed genes. Taf4 is therefore required for *de novo* PIC formation necessary for activation of neurogenic genes and for efficient dissociation of the PIC at pluripotency genes.

### Taf4 is essential for contractile cardiomyocyte differentiation

In the spontaneous differentiation experiments, expression of the early cardiac markers *Nkx2–5* and *Anf* was reduced and in accordance with this, we observed a lack of spontaneously contractile cardiomyocytes from the *Taf4a*^−/−^ EBs, whereas contractile cardiomyocytes were frequently seen from WT EBs. To specifically examine cardiomyocyte differentiation, EBs were cultured for 5 days in medium containing 20% FCS, before plating on gelatin-coated plates for a further 9 days.

**Figure 8:**
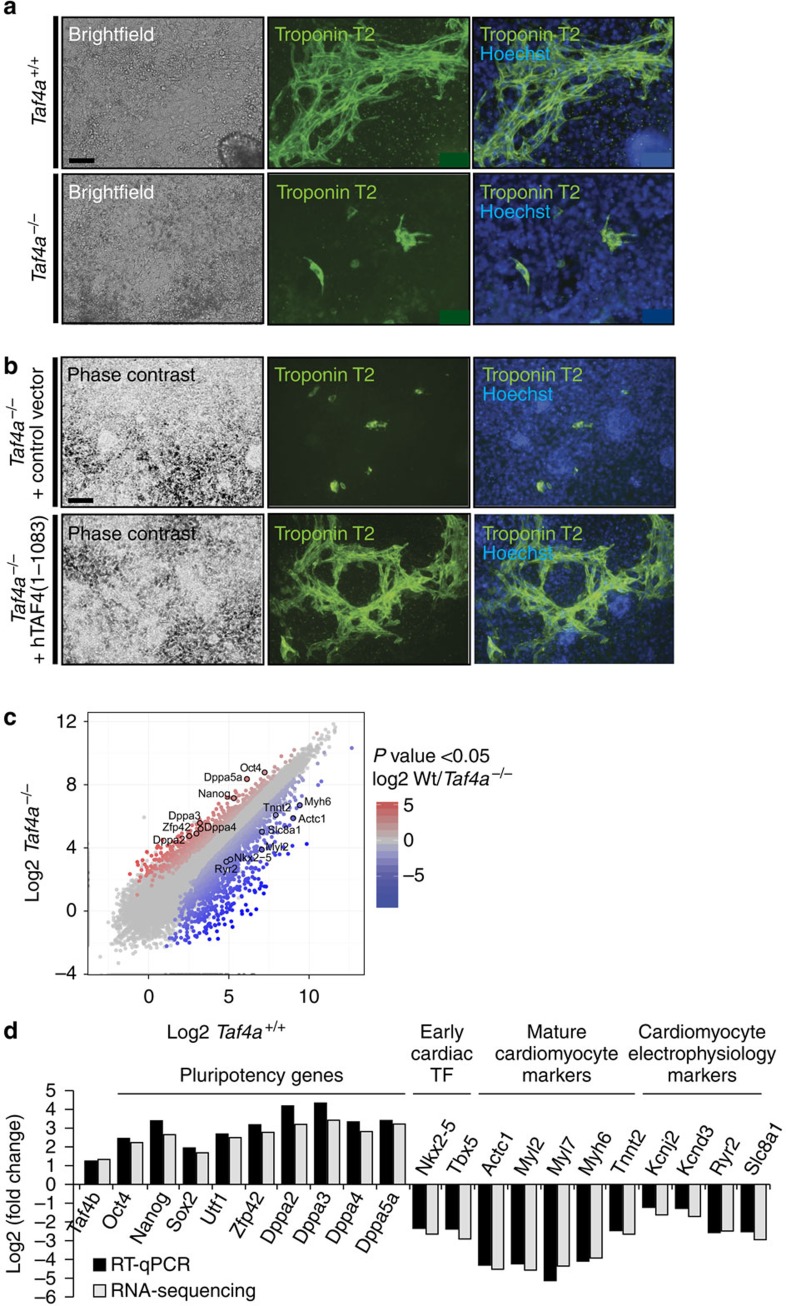
Taf4 is required for cardiomyocyte differentiation. (**a**) Phase-contrast microscopy and Tnnt2 labelling of EBs differentiated into cardiomyocytes 9 days after plating. (**b**) Phase-contrast microscopy and Tnnt2 labelling of ES cells engineered to re-express exogenous Taf4 differentiated into cardiomyocytes 9 days after plating. All images were taken at × 20 magnification. (**c**) Global comparison of RNA-seq data in *Taf4a*^−/−^ compared with WT at the EB8 stage. The expression of several cardiomyocyte markers and pluripotency genes are highlighted. (**d**) Comparison of the changes in expression of selected marker genes measured by RNA-seq and by RR–qPCR. Scale bars 50 μm.

Spontaneously contracting cardiomyocytes were readily identified around 3–5 days after plating of WT EBs, whereas no contractile activity of mutant cells was observed during the 9-day period. In agreement with this, Tnnt2 immunostaining revealed formation of well-defined sarcomeric structures in the differentiated WT cells, whereas such structures were severely reduced, disorganized and patchy in the *Taf4a*^−/−^ cells (Fig. 8a). Importantly, re-expression of full-length Taf4 restored proper Tnnt2 expression and formation of organized and functional sarcomeric structures restoring observable contractile areas (Fig. 8b, Supplementary Fig. 10A). Taf4 is therefore essential for differentiation of contractile cardiomyocytes *in vitro*.

RNA-seq from day 9 differentiated WT and *Taf4a*^−/−^ ESCs identified 636 upregulated genes and 1,550 downregulated genes (Fig. 8c,d) associated with muscle contraction, mesoderm development, cell adhesion and signal transduction (Supplementary Fig. 10B). Expression of many early and late cardiomyocyte markers, including those involved in electrophysiology of contraction, were strongly reduced in mutant cells (Fig. 8d and Supplementary Fig. 10B). Nevertheless, as seen above, pluripotency gene expression was not properly repressed during cardiomyocyte differentiation. Taf4 is therefore required for normal activation of the gene expression programme required for differentiation of functional cardiomyocytes.

## Discussion

We define an essential role of Taf4 in mouse embryogenesis. Previous inactivation of Tbp or ubiquitous core Tafs led to pre-implantation or early post-implantation embryonic death^35^,^36^,^37^,^38^. Due to this early lethality, TAF function at later stages of mammalian embryogenesis where complex lineage specification and differentiation processes occur is so far unknown. In contrast, *Taf4a*^−/−^ embryos survive until E9.5 and display many developmental defects. The longer survival of these embryos may be explained by redundancy with Taf4b that maintains TFIID integrity and all functions required for early development and gastrulation, after which the essential role of Taf4 in specific developmental processes can be observed. This idea is supported by the overlapping expression of *Taf4a* and *Taf4b* at early stages of development, the expression of *Taf4b* in the *Taf4a*^−/−^ embryos and more directly by our observation that Taf4 and Taf4b are redundant for ESC viability. Moreover, *Taf4b*^−/−^ mice are viable and show defects only at the adult stage^7^,^39^. Thus, while Taf4 can fully replace Taf4b during embryogenesis, Taf4b can compensate for Taf4 loss in ESCs and at early, but not later stages of embryogenesis. The observed redundancy may reflect the integration of Taf4b in TFIID in *Taf4a*^−/−^ ESCs to maintain its integrity. However, we cannot exclude other interpretations such as the presence of Taf4b in an alternative TFIID-like complex functioning during early development, and a requirement for Taf4-containing TFIID only at later stages to regulate expression of a distinct subset of genes that govern further development.

*Taf4a*^−/−^ embryos are characterized by several defects. Embryos displayed an overall atrophy resulting in part from increased apoptosis, the absence of a large trunk region and a strongly reduced forebrain domain. However, a striking feature is the absence of foregut invagination associated with lack of gut tube formation and aberrant ventral morphogenesis. In WT embryos, ventral folding morphogenesis achieves gut internalization, proper ventral localization of the heart and enclosure of the fetus in extraembryonic membranes^40^. *Taf4a*^−/−^ embryos are characterized by incomplete enclosure in the yolk sac, misplacement of the heart that develops anterior to the head and a lack of embryo turning. This aspect of the phenotype strongly resembles embryos lacking Bmp2 that arrest before turning and show many similar features in particular misplacement of the heart anterior to the head^40^. Analysis of *Taf4a*^−/−^ embryos thus confirms the importance of correct ventral morphogenesis in positioning of head and heart. Nevertheless, Taf4 is not required for the critical visceral endoderm expression of Bmp2 that persists in mutant embryos, suggesting that it may rather be required for the downstream transcriptional response of the Bmp2 pathway.

While the above phenotypes highlight processes defective in *Taf4a*^−/−^ embryos, others occur almost normally. Taf4 is dispensible for many patterning events, activation of early neural and mesodermal makers and the posterior development of the embryo that, in contrast to the anterior, is almost normal. Importantly, Pou5f1-expressing PGCs are formed in *Taf4a*^−/−^ embryos although they cannot migrate properly due to the absence of a developed hindgut. Generation of PGCs involves complex transcriptional and epigenetic reprogramming events including reactivation of pluripotency genes as well as changes in histone and DNA methylation^41^,^42^,^43^. Taf4 is therefore not required for this naturally induced reprogramming.

This observation contrasts with a reported role of Taf4 in maintaining ESC pluripotency and promoting induced pluripotent stem cell reprogramming *in vitro*. Pijnappel *et al*.^27^, reported that Taf4 overexpression enhanced the ability of Pou5f1, Nanog, Sox2 and Myc to reprogram human and mouse fibroblasts to induced pluripotent stem cells *in vitro*. Furthermore, they reported that Taf4 or Taf4b short hairpin RNA knockdown resulted in spontaneous differentiation of ESCs implying that both are essential for maintaining stemness. They further proposed Taf4 as an integral component of the pluripotency network as Pou5f1 and Nanog bind a critical enhancer driving high *Taf4a* expression in ESCs. In contrast, Bahat *et al*.^44^ used siRNA knockdown and reported that Taf4b plays a critical role in maintaining ES cell stemness, whereas Taf4 knockdown had no effect. Thus, there are conflicting data on the role of Taf4 and Taf4b in maintaining ESC pluripotency, perhaps related to the techniques used or the efficiencies of knockdown achieved.

Our use of genetically modified mice and ESCs clearly shows that Taf4 is dispensible for pre-implantation development *in vivo* and that *Taf4a*^−/−^ ESCs are almost indistinguishable from WT ESCs *in vitro* when grown under pluripotent conditions. As *Taf4b*^−/−^ mice are viable, genetics indicates that neither Taf4 nor Taf4b are essential for pluripotency, but rather are redundant for this function. Moreover the appearance of PGCs in the *Taf4a*^−/−^ embryos indicates that Taf4b can also replace Taf4 in this reprogramming event.

Rather than playing a role in ESC pluripotency, our data show that Taf4 acts during differentiation. Similar to what is seen *in vivo*, *Taf4a*^−/−^ EBs initiate expression of early markers of all three germ layers, but Taf4 is essential for the activation of later stage differentiation genes. Analysis of RA-driven neurogenic differentiation of *Taf4a*^−/−^ ESCs confirmed that expression of early-induced genes is affected less than later markers. The activation kinetics reflect somewhat different regulatory mechanisms as promoters of early-induced genes show appreciable PIC formation in the undifferentiated state that is increased during differentiation, whereas late-induced genes show little PIC formation in ESCs, their activation being almost completely dependent on *de novo* PIC formation. In mutant ESCs reduced PIC formation is seen at early genes, but is not increased during differentiation, and *de novo* PIC formation at later genes is almost completely abolished. Thus while Taf4b-containing TFIID can assemble the PIC in ESCs, Taf4 is critical for *de novo* PIC formation on later stage neurogenic genes.

The defective neuronal and cardiomyocyte differentiation of *Taf4a*^−/−^ ESCs is reminiscent of the role of Taf3 in endodermal differentiation of ESCs^45^. Short hairpin RNA silencing showed that Taf3 is not required for ESC pluripotency, but plays a specific role in endoderm differentiation. Here we show that Taf4 is not required for ESC pluripotency, but plays a more general role in differentiation of all three germ layers. It is possible that the overall reduced amounts of TFIID in *Taf4a*^−/−^ ESCs may impact their differentiation. Pijnappel *et al*.^27^ (and Fig. 5a in this study) showed higher levels of Tbp and TAFs in ESCs compared with fibroblasts and suggested that high TFIID levels are required for pluripotency. Reduced TFIID would therefore be expected to affect pluripotency rather than differentiation. For this reason, we postulate that the defective differentiation of *Taf4a*^−/−^ ESCs and the defects seen in Taf4-null embryos reflect loss of a specific function of Taf4 rather than an overall decrease in TFIID.

Bahat *et al*.^44^ reported that Taf4 knockdown rendered ESCs refractory to the effects of RA, whereas we show here incomplete differentiation of RA-treated *Taf4a*^−/−^ ESCs. However, Bahat *et al*.^44^ treated ESCs directly with RA without prior growth as EBs, an essential feature of the neurogenic differentiation protocol. Nevertheless, an interesting feature of differentiating *Taf4a*^−/−^ neurons and cardiomyocytes is the persistent expression of pluripotency genes. Bahat *et al*.^44^ showed that Taf4b, but not Taf4, interacts with Pou5f1. The interactions of Pou5f1 with the Taf4b-containing TFIID in the *Taf4a*^−/−^ ESCs may promote expression of the pluripotency network to the detriment of redistribution to differentiation genes thereby explaining the persistent expression of pluripotency genes in absence of Taf4.

## Methods

### Recombinant mice

C57Bl6J mice carrying *Taf4a* null alleles where exons 11 and 12 have been deleted were previously described^16^. *Taf4a*^−/−^ embryos were derived from crosses of 6 to 7-week-old *Taf4a*^+/-^ animals. Mice were kept in accordance with institutional guidelines regarding the care and use of laboratory animals and in accordance with National Animal Care Guidelines (European Commission directive 86/609/CEE; French decree no.87–848). All procedures were approved by the French national ethics committee.

### Histology and immunohistochemistry

Deciduae were fixed in Bouin's fluid for 14 h before being dehydrated and embedded in paraffin. The embedded decidua were cut in 5-μm sections and stained with hematoxylin and eosin. Other sections were used for terminal deoxynucleotidyl nick-end labelling assay using the ApopTag Peroxidase *In Situ*.

 Apoptosis Detection Kit (Millipore) as described by the manufacturer. To analyse proliferation, pregnant females were peritoneal injected with BrdU (50 mg kg^−1^ final) and sacrificed after 90 min. Paraffin sections on deciduae obtained from these litters were boiled in citrate buffer for antigen retrieval and staining was performed with primary anti-BrdU (555627, BD Biosciences). Pecam staining on whole-mount embryos and yolk sacs was performed as described^46^.

### Whole-mount *in situ* hybridization

Mouse embryos were dissected in ice-cold PBS and fixed O/N in 4% paraformaldehyde (PFA)/PBS. After several washes in PBS-0.1% Tween-20 (PBT) to eliminate traces of formaldehyde, embryos were bleached for 1 h in 3% H_2_O_2_/PBT and washed 3 × 5 min in PBT before being digested with Proteinase K (10 μg ml^−1^) for 30 s, 1 min 30 s or 2 min 30 s (E7.5, E8.5 and E9.5). Digestion was stopped by 5 min incubation in 2 mg ml^−1^ glycine/PBT. Embryos were washed again 3 × 5 min in PBT before post-fixing for 20 min in 0.2% glutaraldehyde/4% PFA/PBS. After further washes they were incubated in prewarmed hybridization buffer and prehybridized for 2 h at 65 °C. The buffer was then replaced with fresh prewarmed hybridization buffer containing the digoxigenin labelled RNA probes and incubated O/N at 65 °C. The next day, embryos were washed twice in the hybridization buffer at 65 °C before treating them with RNaseA to reduce background. The embryos were then blocked for 2 h in 2% blocking solution (Roche)/TBS-0.1% Tween-20 (TBST) and incubated O/N in the same solution containing 1:2,500 anti-digoxigenin antibody (Roche). The next day the embryos were washed in TBST, before washing them in NTMT buffer (2 × 10 min) and developing the signal with BM Purple (Roche). Most *in situ* hybridisations were performed on three independent WT and mutant embryos.

### Generation, culture and differentiation of *Taf4a* ^−/−^ ES cells

*Taf4a*^+/−^ mice were crossed and E3.5 blastocysts were flushed out the uterus and cultured on mitomycin treated MEF feeders. The ICM outgrowths were trypsinized and passaged on 24-well plates. ES cells were further cultured on inactivated MEF feeders under standard conditions (DMEM 4.5 g l^−1^ glucose/Glutamax supplemented with 15% heat inactivated FCS, LIF, nonessential amino acids and β-mercaptoethanol). *Taf4a*^−/−^ ES cells were identified by PCR as previously described^16^. For spontaneous differentiation, ES cells were feeder depleted and allowed to form EBs in DMEM 4.5 g l^−1^ glucose/Glutamax supplemented with 10% heat inactivated FCS, nonessential amino acids and β-mercaptoethanol in non-adherent bacterial plates. EBs were cultured for a total of 14 days with medium change every other day. Cells were differentiated by RA into neurons as described^33^,^34^. For differentiation into cardiomyocytes, 4 × 10^6^ cells were grown as EBs in EB medium containing 20% heat inactivated FCS and cultured for 5 days. EBs were then transferred to gelatin-coated plates and cultured for 9 more days. For rescue experiments, a linearized control plasmid or a plasmid containing recombinant human or mouse TAF4 complementary DNA (cDNA) was electroporated into *Taf4a*^−/−^ ES cells and selected with puromycin. Resistant clones were further amplified and differentiated as described above.

### Immunostainings and alkaline phosphatase staining

For immunostaining, cells were fixed for 30 min in 4% paraformaldehyde at room temperature, followed by incubation in blocking solution (PBS, 0.1% Triton X-100, 5% normal goat serum) for at least 2 h. Primary antibodies were incubated O/N in blocking solution and then washed 3 times with PBS, 0.1% Triton X-100 for 10 min per step to reduce unspecific binding before incubation with secondary antibodies. The Alkaline Phosphatase Detection Kit (SCR004; Millipore) was used following the manufacturers instructions.

### Cell growth and cell cycle analysis

To determine growth rates, 5 × 104 cells from either WT or mutant ES cells, were plated gelatin-coated six-well plates in triplicate and counted for 4 consecutive days. For cell cycle analysis, cells were fixed in 70% ice-cold ethanol O/N, washed with PBS and then stained with propidium iodide for 4 h. Fluorescence was detected using the BD FACSCalibur flow cytometer and the obtained data were analysed with the CellQuest software.

### Gene expression analyses

Total RNA from ES cells or EBs was isolated using the GenElute Mammalian Total RNA Miniprep Kit (Sigma-Aldrich). cDNA was generated using Maxima First Strand cDNA Synthesis Kit for RT-qPCR (Thermo Scientific), following the manufacture's instructions. PCR used the SYBR Green PCR Master Mix from Qiagen and the LightCycler 480 Real-Time PCR-System (Roche). Relative expression levels were normalized to GAPDH. RNA-seq analyses were performed essentially as described^47^,^48^. All RT-qPCR experiments were performed in triplicate. Messanger RNA-seq was performed essentially as previously described^48^. Sequence reads mapped to reference genome mm9/NCBI37 using Tophat^49^. Data normalization and quantification of gene expression was performed using the DESeq 2 Bioconductor package^50^. Two biological replicates from two independent WT and *Taf4a*^−/−^ ESC lines were analysed. Gene ontology analyses were performed using the functional annotation clustering function of DAVID (http://david.abcc.ncifcrf.gov/).

### Preparation of ES cell nuclear extracts, IP and immunoblot

ES cells were resuspended in swelling buffer (25 mM Tris pH 7,9, 10 mM KCl, 1.5 mM MgCl2, 1 mM DTT), incubated for 10 min on ice, lysed using a dounce homogenizer and centrifuged. Pelleted nuclei were resuspended in extraction buffer (50 mM Tris pH 7,5, 400 mM NaCl, 0.5% NP-40, 5% Glycerol and 1 × Roche complete protease inhibitor) and proteins were extracted for 2 h at 4 °C with rotation. For IP, 500 μg nuclear extract were pre-cleared for 2 h with 50 μl of protein G sepharose beads, before addition of with 1 μg of anti-TBP (3G3) antibody O/N at 4 °C. The next day, bound complexes were recovered by incubating the extract with 25 μl protein G sepharose beads for 2 h at 4 °C. Beads containing bound proteins were washed three times with extraction buffer for 15 min per wash and once with the buffer containing 150 mM NaCl before being boiled in in SDS loading buffer for 5 min at 95 °C. Bound proteins were analysed by Western blot using the indicated antibodies. A control IP for each extract was performed in parallel using a non-specific antibody against glutathione-S transferase (GST). Uncropped western blots are provided for all immunoblots in Supplementary Figure 11.

### Gel filtration

500 μl of nuclear extracts prepared as described above containing 6 mg of protein were injected onto a equilibrated Superose 6 (10/300) column and run at 0.4 ml min^−1^. 500 μl fractions were collected and analysed by western blot.

### Antibodies

The following antibodies were used: anti-OCT4 (sc-9081; Santa Cruz), anti-NANOG (RCAB001P; Reprocell), SOX2 (L1D6A2; Cell Signaling), previously described or recently developed in house antibodies against TBP (3G3), TAF1, TAF3 (ref. 15), TAF4 (32TA and also sc-136093; Santa Cruz), TAF4B (ref. 16), TAF5 (1TA), TAF6 (25TA), TAF7 (19TA), TAF12 (22TA), and TAF13 (16TA), TAF10 (sc-102125; Santa Cruz) and in house 6TA2B11 (ref. 51), anti-Pol II (sc-9001x; Santa Cruz), anti-Tnnt2 (MS-295-P1; Thermo scientific). We also used a polyclonal TAF4B antibody (2057) raised against a peptide corresponding to amino acids 692–710 of human TAF4B that is 100% conserved in mouse Taf4b. This antibody also recognizes Taf4 as this region comprises only two amino acid differences with Taf4b. The following antibody dilutions were used for immunostaining. Pou5f1 and Nanog, 1:100; Sox2, and Taf4, 1:50; Tubb3 and Tnnt2, 1:1,000. For immunoblots all antibodies were diluted 1:1,000 except polyclonal serum 2057 that was diluted 1:2,000.

### Chromatin IP sequencing

Chromatin IP experiments were performed on 0,4% PFA-fixed chromatin according to standard protocols as previously described^15^. Chromatin IP sequencing libraries were prepared and sequenced on an Illumina Hi-seq2500 as single-end 50-base reads. After sequencing, peak detection was performed using the MACS software (ref. 52 http://liulab.dfci. harvard.edu/MACS/). Global clustering, meta analyses and quantitative comparisons were performed using seqMINER^53^ and R (http://www.r-project.org/).

## Additional information

**Accession code:** The RNA-seq data have been deposited in the GEO database under accession code GSE70726 and the ChIP-seq data under the accession code GSE70661.

**How to cite this article:** Langer, D. *et al*. Essential role of the TFIID subunit TAF4 in murine embryogenesis and embryonic stem cell differentiation. *Nat. Commun.* 7:11063 doi: 10.1038/ncomms11063 (2016).

## Supplementary Material

Supplementary Information Supplementary Figures 1-11 and Supplementary Table 1.

## Figures and Tables

**Table 1:** Quantification of observed phenotypes in *Taf4a* ^−/−^ embryos.

**Gestational age**	**Cardiac defects**				**Other defects**	
	**No Heart**	**Heart in amniotic cavity**	**Heart in exocoelmic cavity**	**Heart outside visceral yolk sac**	**Allantois not fused**	**Embryo not turned**
E8.5	22/24 (92%)	ND	ND	ND	NA	NA
E9.5	18/66 (27%)	1/25 (4%)	21/25 (84%)	3/25 (12%)	19/21 (90%)	66/66 (100%)

ND, not determined; NA, not applicable
