# Supplementary material for: Essential role of the TFIID subunit TAF4 in murine embryogenesis and embryonic stem cell differentiation
Source: Nat Commun. 2016 Mar 30;7:11063. doi: 10.1038/ncomms11063 (PMC4820908; doi:10.1038/ncomms11063)
Supplement: Supplementary Information — Supplementary Figures 1-11 and Supplementary Table 1. [file ncomms11063-s1.pdf]

Supplementary Figures.

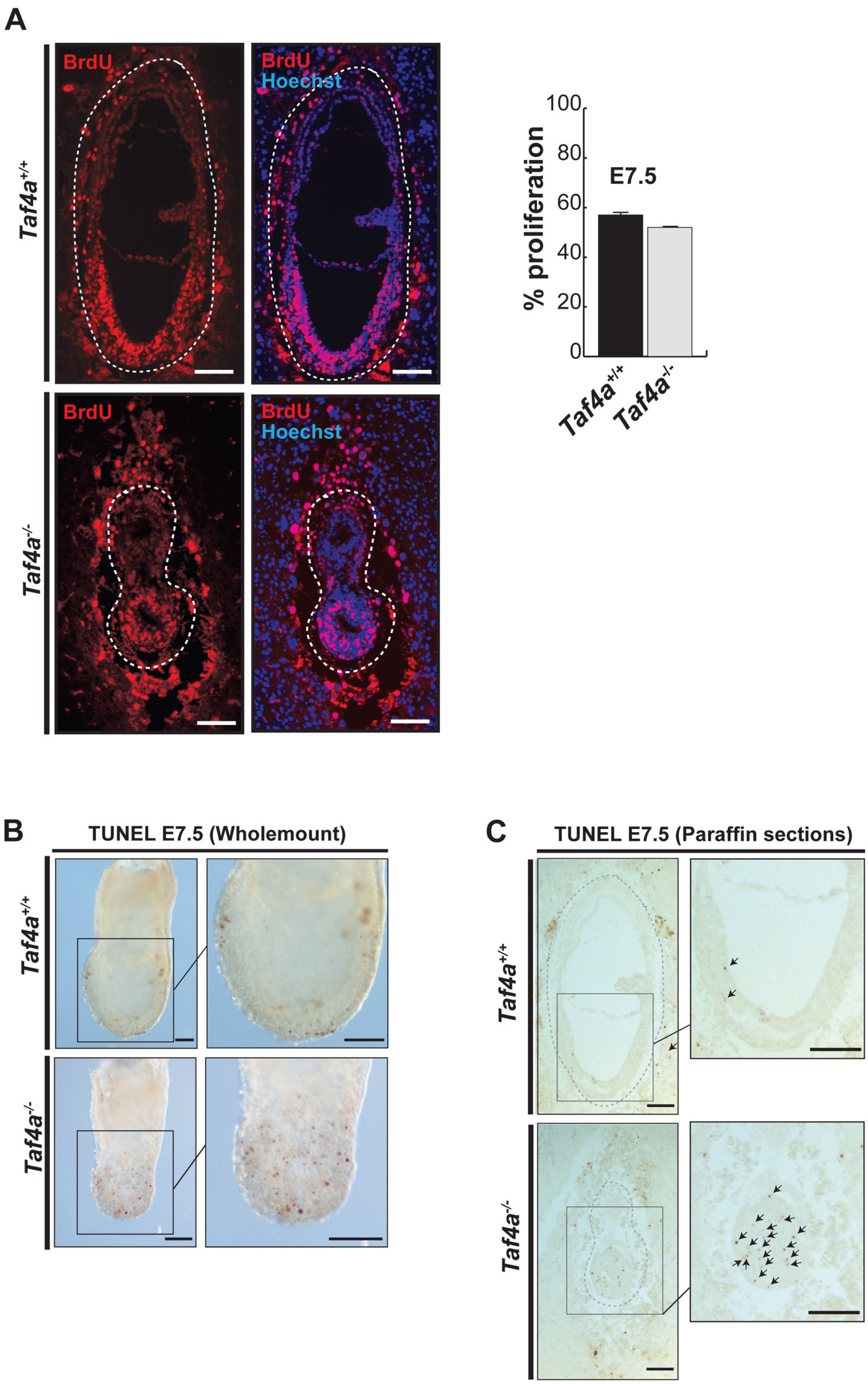

Supplementary Fig. 1

Supplementary Figure 1. Related to Figure 1. Enhanced apoptosis in *Taf4a*$^{-/-}$ embryos. **A.** BrdU incorporation in WT and mutant embryos was determined at E7.5 and the mitotic index was quantified. Represented as a mean with error bars showing standard deviation (± SD) using the Excel STDEV.P function (N=3). **B-C.** TUNEL assay in whole mount embryos or on paraffin sections (C) reveal significantly elevated apoptosis rates in *Taf4a*$^{-/-}$ embryos. Apoptotic cells are highlighted with black arrows in panel C. Scale bars: 100 µm in all panels.

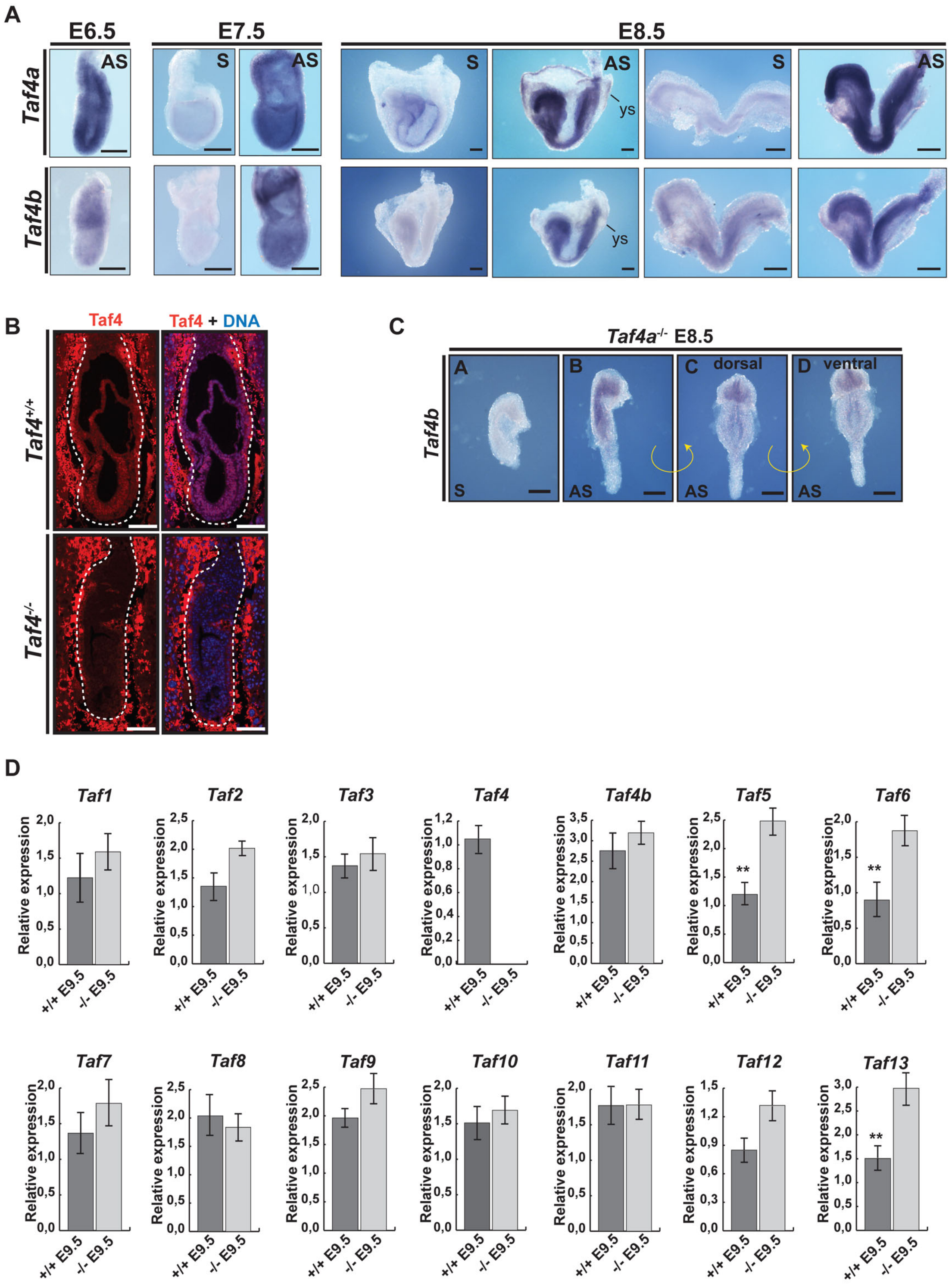

Supplementary Fig. 2

Supplementary Figure 2. Related to Figure 1. Overlapping Taf4a and Taf4b expression during embryonic development. **A.** *In situ* hybridisation with sense (S) and anti-sense (AS) *Taf4a* or *Taf4b* specific probes on E6.5, E7.5 and E8.5 embryos. **B.** Immunostaining of E7.5

WT and mutant embryo sections with antibody against Taf4. **C.** *In situ* hybridisation to reveal *Taf4b* expression in *Taf4a*$^{-/-}$ embryos at E8.5. **D.** Relative expression of Tafs in WT and mutant embryos at E9.5 detected by quantitative RT-PCR analysis. Data were normalized to expression of Tbp and are represented as mean ± SD (n=4). Scale bars: 100 μm in all panels.

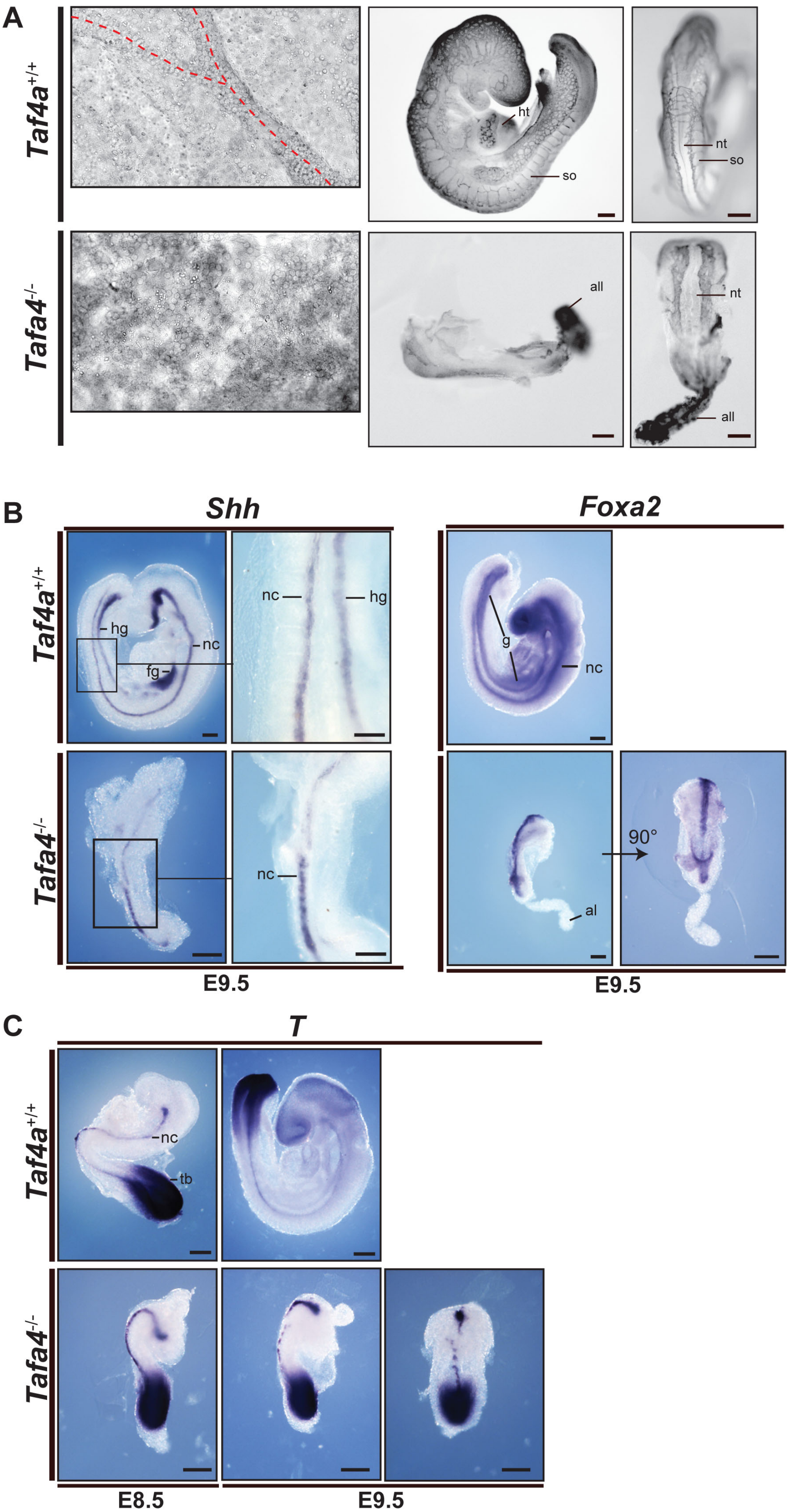

Supplementary Fig. 3

Supplementary figure 3. Related to Figure 2. *Taf4a*$^{-/-}$ embryos exhibit defects in development of vasculature, axis elongation and gut tube formation. **A.** Development of vasculature in the yolk sac (left panels) and embryo proper (middle (sagittal view) and right (dorsal view) panels at E9.5 was determined by immunostaining with the endothelial marker Cd31 (Pecam-1). **B.** Development of anterior-posterior axis and gut were examined by *in situ* hybridisation with probes for *Shh* and *Foxa2* at E9.5. **C.** *In situ* hybridisation for *T* (brachyury) confirmed that the stained discontinuous and irregular structure represents the notochord. Scale bars: 100 µm in all panels.

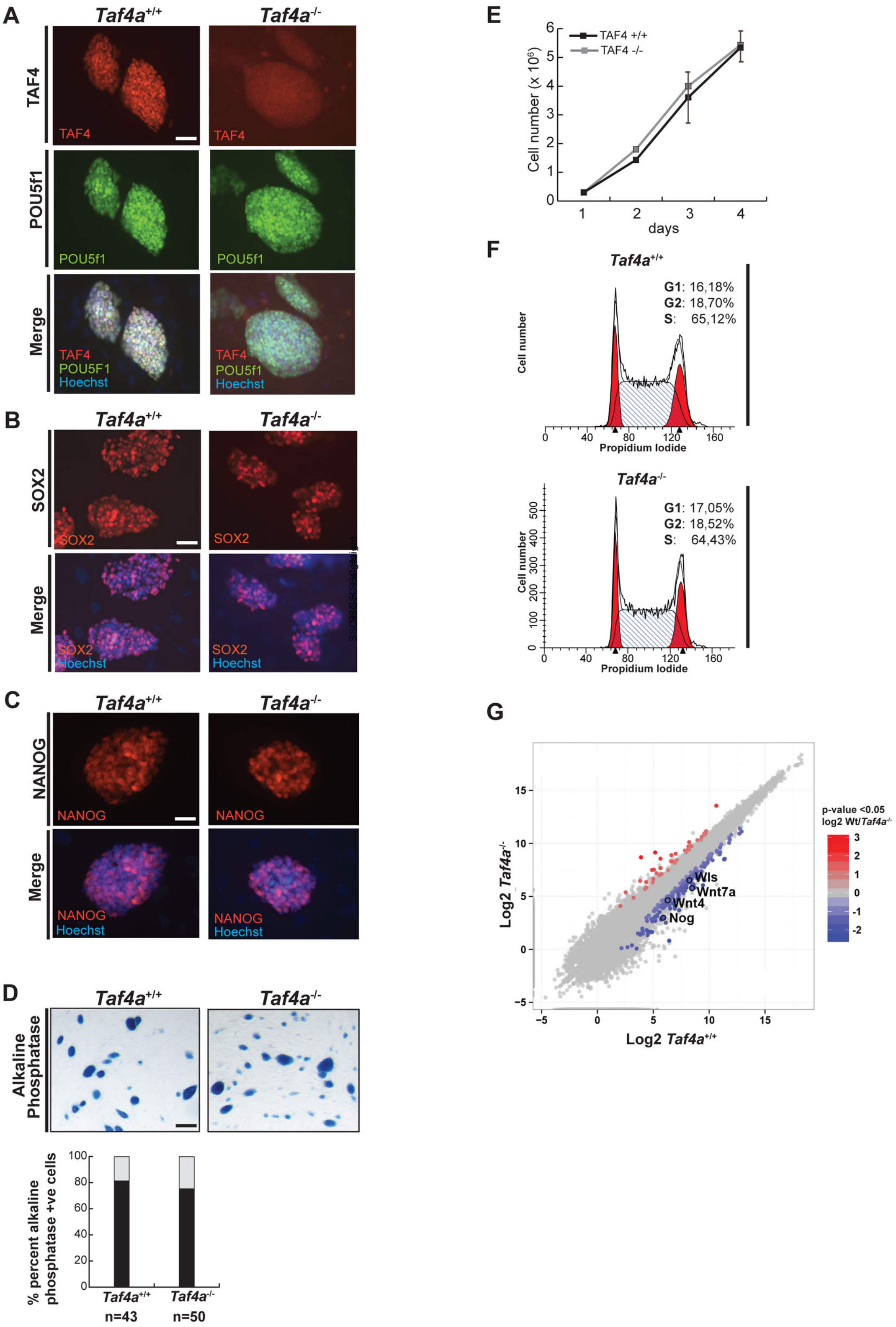

Supplementary Fig. 4

Supplementary figure 4. Related to Figure 5. Characterization of WT and *Taf4a*$^{-/-}$ ES cells. **A-C.** Immunostainings reveal expression of the pluripotency factors Oct4 (A), Sox2 (B) as well as Nanog (C) in the absence of Taf4. **D.** Comparable alkaline phosphatase staining in

WT and *Taf4a*$^{-/-}$ ES cells. Scale bars: 100 µm in panel A, 50 µm in panels B and C. **E.** Comparable growth of WT and *Taf4a*$^{-/-}$ ES cells over a period of 4 days. The average values ± SD of three experiments are shown. **F.** Cell cycle profiles were analyzed by FACS analysis of propidium iodide stained WT and *Taf4a*$^{-/-}$ ES cells. **G**. Global comparison of gene expression in two WT vs *Taf4a*$^{-/-}$ ESC lines. While the overall gene expression profile is highly similar, several genes showing minor changes in expression are highlighted.

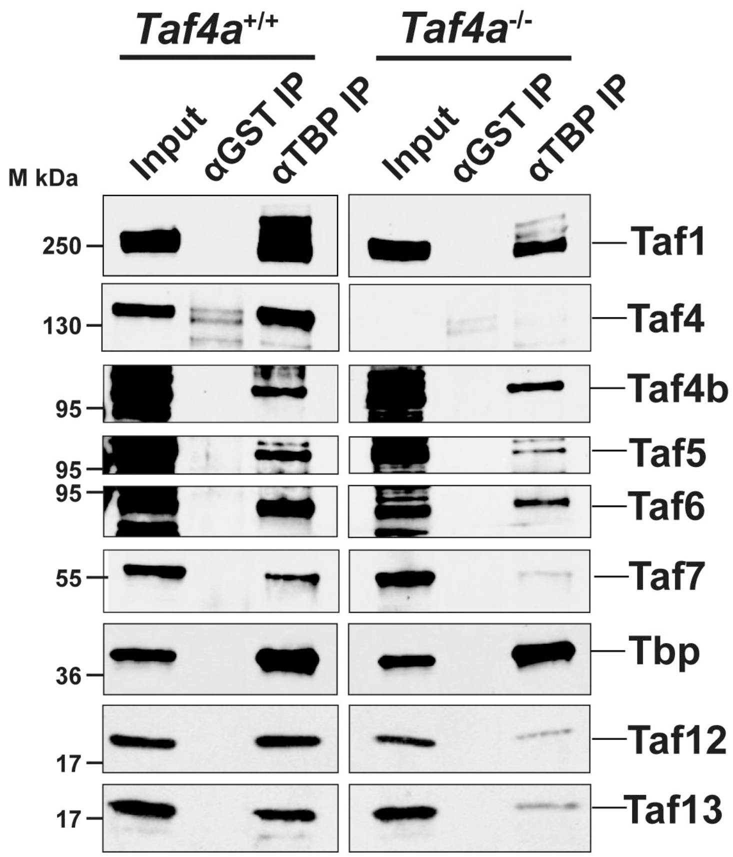

Supplementary Fig S5

Supplementary figure 5. Related to Figure 5. Incorporation of Taf4b into TFIID. Extracts from WT and Taf4-mutant ESCs were subjected to immunoprecipitation with anti-TBP antibody and the presence of Tbp and Tafs detected in the precipitated fractions by immunoblots. GST antibody was used for a control immunoprecipitation. The positions of the molecular mass markers are indicated.

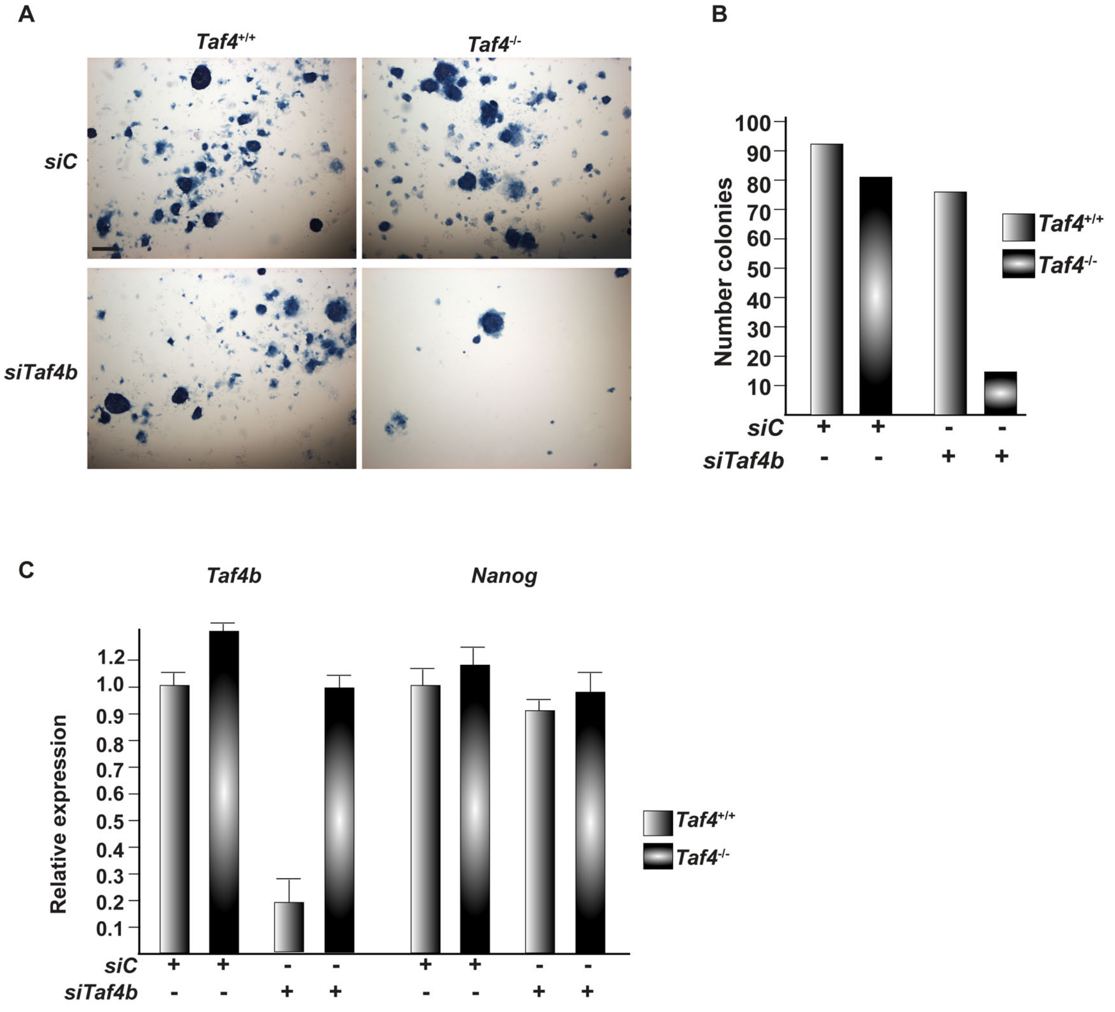

Supplementary Fig. 6

Supplementary figure 6. Related to Figure 5. Redundant functions of Taf4 and Taf4b. WT or *Taf4a*$^{-/-}$ cells were transfected with control siRNA (siC) or the Dharmacon siRNA pool directed against Taf4b. Following transfections cells were re-plated and grown for 5 days before staining with alkaline phosphatase or preparation of RNA. **A.** Bright field images of cells transfected with the indicated siRNAs. Scale bar: 100 µm. **B.** Total numbers of colonies obtained under each condition. **C.** RT-qPCR measurement of *Taf4b* and *Nanog* expression under the different conditions. Data represented as mean ± SD (n=3).

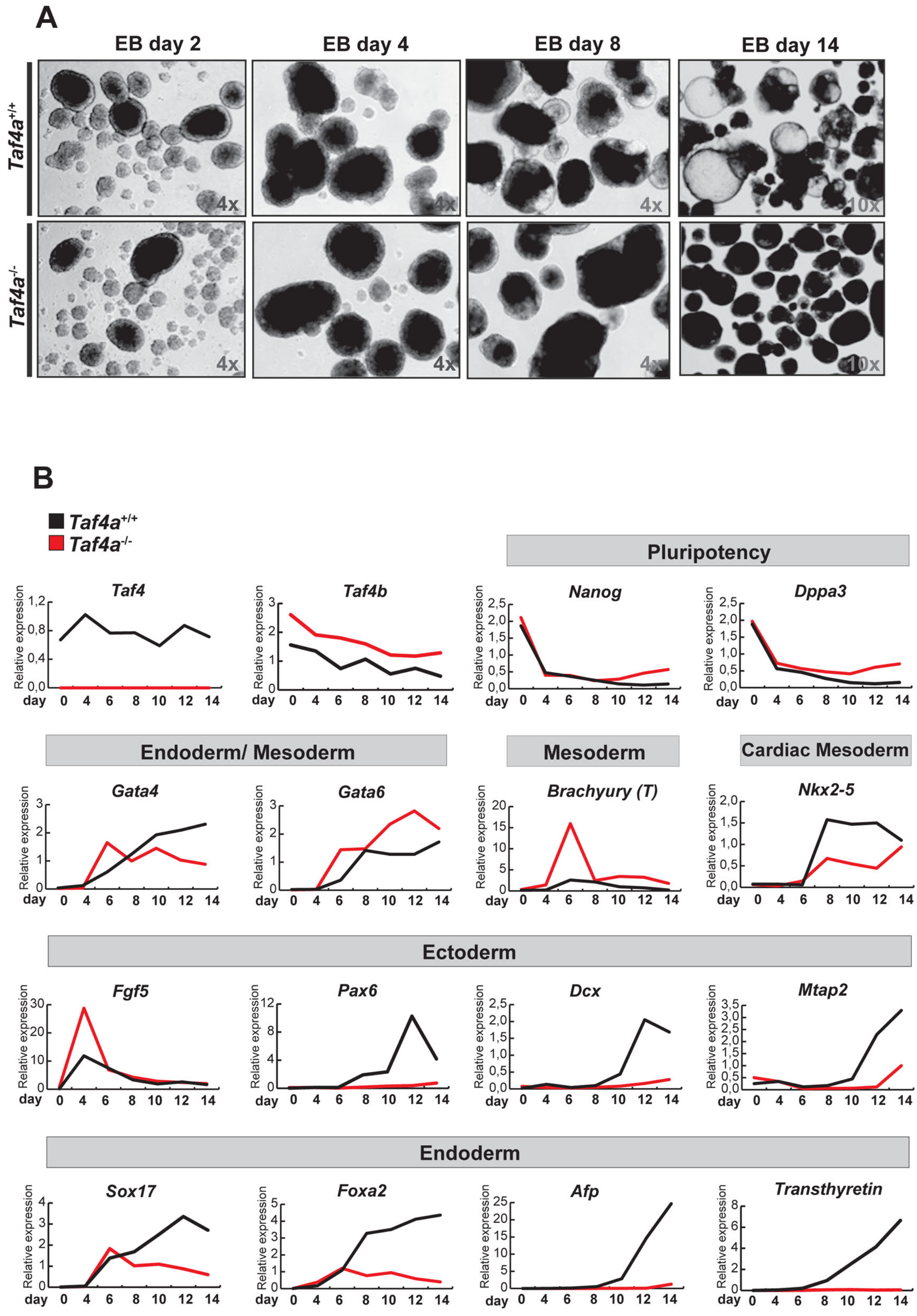

Supplementary Fig. 7

Supplementary figure 7. Related to Figure 5. *Taf4a*$^{-/-}$ ES cells fail to properly differentiate *in vitro*. **A**. Morphology of WT and *Taf4a*$^{-/-}$ ES cells cultured as embryoid bodies in the absence of LIF over a period of 14 days. **B.** Quantitative RT-PCR analysis of differentiation markers

expression at several time points during *in vitro* differentiation reveal the failure of *Taf4a*$^{-/-}$ ES cells to properly up-regulate expression of cardiac mesodermal, ectodermal and endodermal markers and to completely down-regulate expression of pluripotency markers. Expression levels were normalized to *Gapdh*.

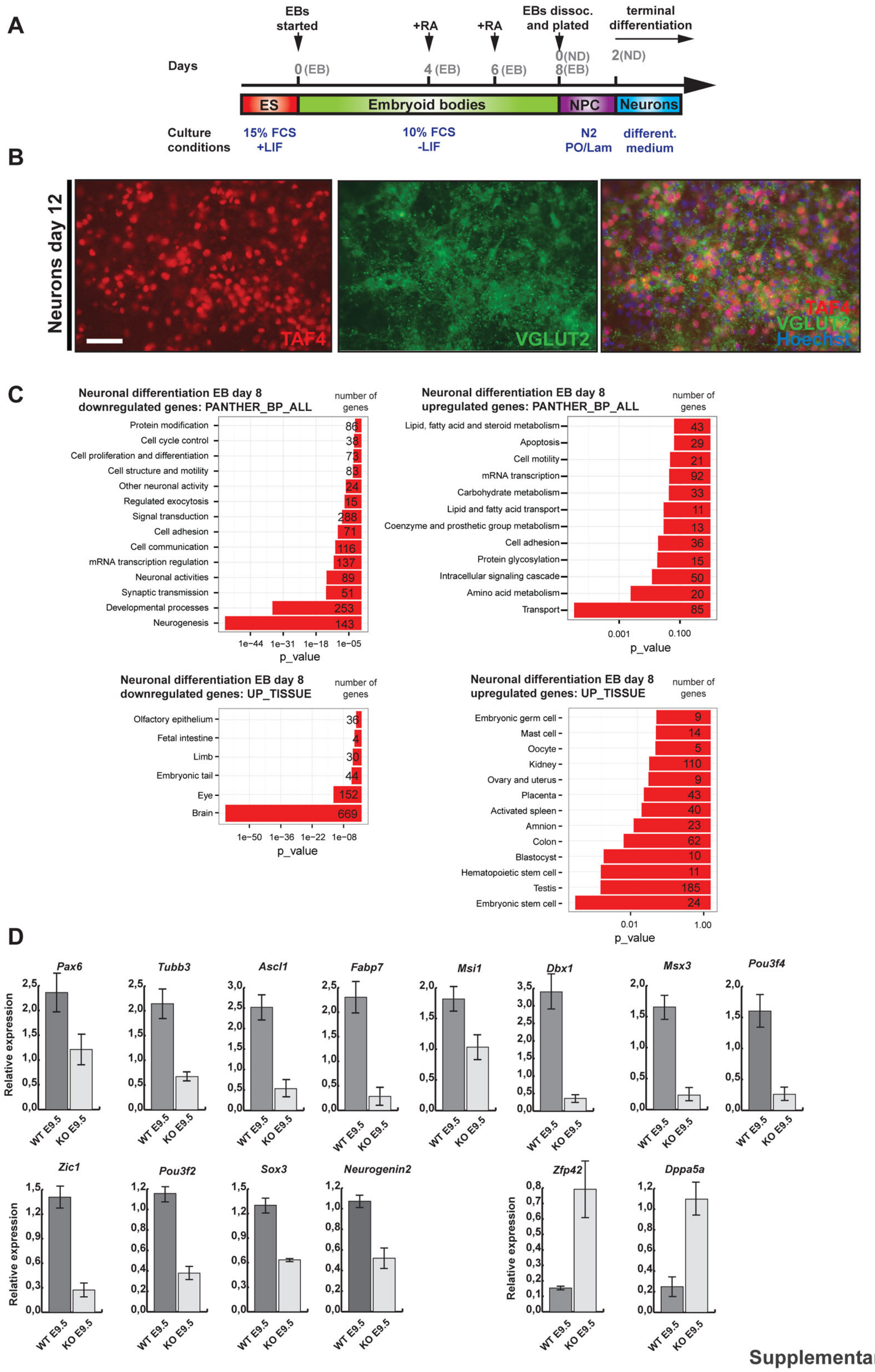

Supplementary Fig. 8

Supplementary figure 8. Related to Figure 6. Differentiation of ES cells into glutamatergic neurons *in vitro*. **A.** Overview of the neuronal differentiation protocol applied to control and *Taf4a*$^{-/-}$ ES cells. **B.** Neurons obtained from WT ES cells were cultured for 12 days and stained for the glutamatergic marker Vglut2 as well as Taf4. **C.** Ontology analysis of genes whose expression is deregulated during neurogenic differentiation of *Taf4a*$^{-/-}$ ES cells. The number of genes in each category is shown. **D.** Expression of neurogenic genes in WT and *Taf4a*$^{-/-}$ E9.5 embryos was measured by RT-qPCR. Data represented as mean ± SD (n=3).

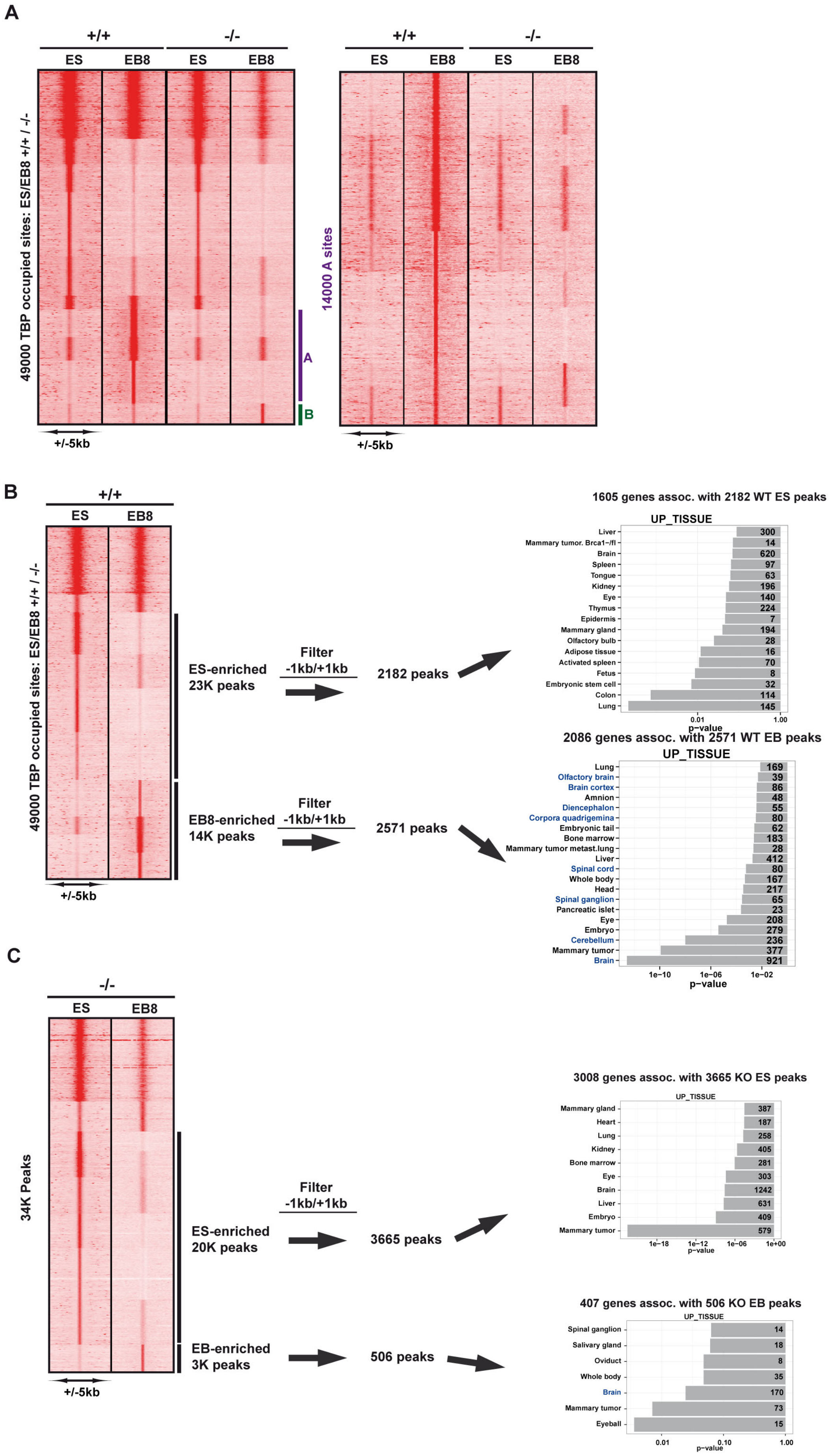

Supplementary Fig. 9

Supplementary figure 9. Related to Figure 7. Relocalisation of Tbp during neuronal differentiation. **A.** Read density cluster analysis of Tbp ChIP-seq at the indicated times in differentiating WT and *Taf4a*$^{-/-}$ ES cells. Right panel shows re-clustering of cluster A of left panel. **B.** Tbp is re-localised to neurogenic genes during differentiation of WT cells. Left panel shows the Read density cluster analysis and the ontology analysis of the genes with enriched Tbp at their transcription start site (TSS) in ES or EB8 is shown. **C.** Tbp is enriched at ES expressed genes during neurogenic genes during differentiation of *Taf4a*$^{-/-}$ ES cells. Left panel shows the Read density cluster analysis and the ontology analysis of the genes with enriched Tbp at their TSS in ES or EB8 is shown.

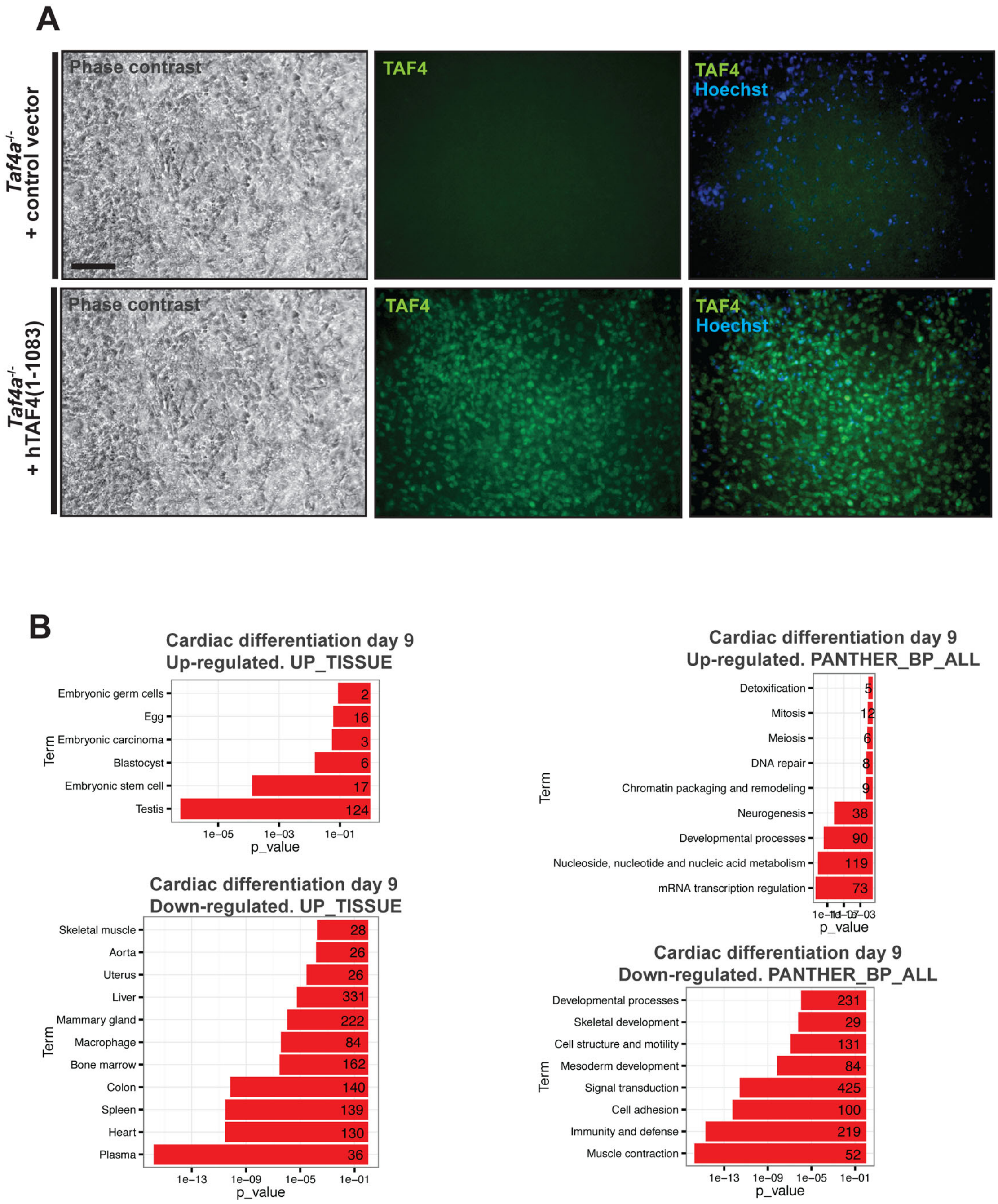

Supplementary Fig. 10

Supplementary figure 10. Related to Figure 8. Cardiomyocyte differentiation of ES cells engineered to re-express exogenous Taf4. **A.** Immunostaining detects Taf4 expression in *Taf4a*$^{-/-}$ ES cells expressing exogenous Taf4 differentiated as cardiomyocytes. Scale bar: 100

μm. **B.** Ontology analysis of genes whose expression is deregulated during cardiomyocyte differentiation of *Taf4a*$^{-/-}$ ES cells.

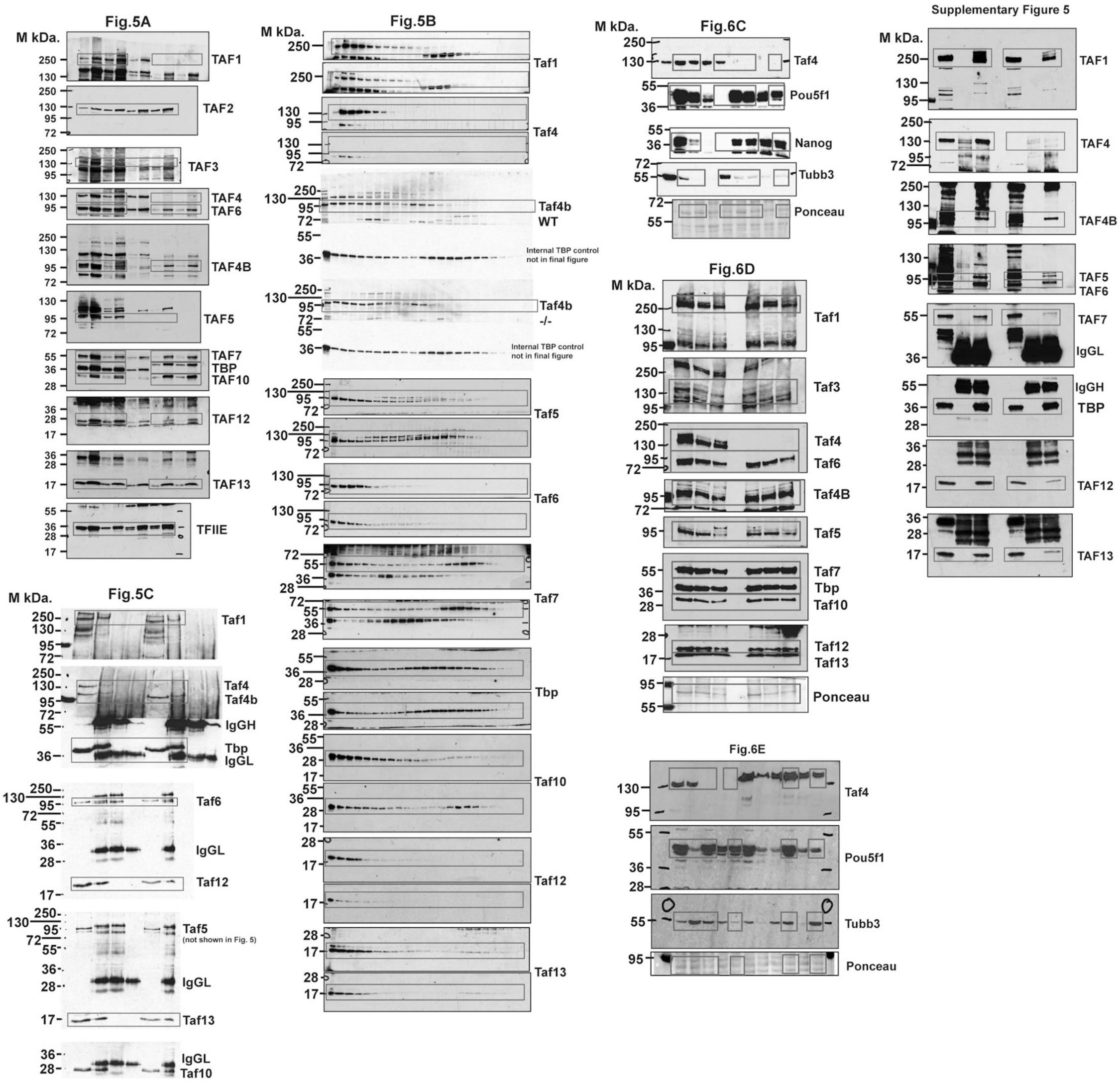

Supplementary Fig. 11

Supplementary figure 11. Uncropped images of Western blots used in main and supplementary figures. The number of the corresponding figure is shown above the panels and the region used to make the figure is boxed. The position of the molecular mass markers is shown.

Gestational age	$Taf4a^{+/+}$	$Taf4a^{+/-}$	$Taf4a^{-/-}$	
E7.5	252 (25.6%)	518 (52.7%)	213 (21.6%)	# mice %
E8.5	245 (26.2%)	43 (46.4%)	255 (27.3%)	# mice %
E9.5	95 (29.4%)	153 (47.3%)	75 (23.2%)	# mice %

Supplementary Table 1

Supplementary table 1. Related to Figure 1. Quantification of embryo genotypes. The table shows the number of embryos with the indicated genotypes of embryos from crosses of *Taf4a*$^{+/-}$ mice at the indicated stages. The total number of mice of each genotype and the % are also shown. *Taf4a*$^{-/-}$ embryos are detected at Mendelian frequency until their death at E9.5.
